# Proteo-metabolomic analysis of fruits reveals molecular insights into variations among Italian Sweet Cherry (*Prunus avium* L.) accessions

**DOI:** 10.3389/fpls.2025.1591996

**Published:** 2025-06-03

**Authors:** Sabrina De Pascale, Antonio Dario Troise, Milena Petriccione, Angelina Nunziata, Danilo Cice, Elvira Ferrara, Andrea Scaloni, Anna Maria Salzano

**Affiliations:** ^1^ Proteomics, Metabolomics and Mass Spectrometry Laboratory, Institute for the Animal Production System in the Mediterranean Environment (ISPAAM), National Research Council, Portici, Italy; ^2^ Research Centre for Olive, Fruit and Citrus Crops, Consiglio per la Ricerca in Agricoltura e l’Analisi dell’Economia Agraria, Caserta, Italy

**Keywords:** sweet cherry, *Prunus avium*, biodiversity, fruit, proteomics, metabolomics

## Abstract

Mass spectrometry-based proteomics and metabolomics tackle the complex interactions between proteins and metabolites in fruits. Independently used to discern phenotypic disparities among plant accessions, these analytical approaches complement well-established DNA fingerprinting methods for assessing genetic variability and hereditary distance. To verify the applicability of integrated proteomic and metabolomic procedures in evaluating phenotypic differences between sweet cherry cultivars, and to potentially relate these findings to specific pomological traits, we conducted a comparative analysis of fruits from ten Italian accessions. We identified 3786 proteins, of which 288 exhibited differential representation between ecotypes, including key components influencing fruit quality and allergenic potential. Furthermore, 64 polyphenols were identified, encompassing anthocyanins, hydroxycinnamic acids, flavanols, hydroxybenzoic acids, flavonols, and flavanones subgroups. Multivariate analysis of total quantitative data outlined cultivar differences and phenotypic relationships. Coherent associations between proteomic and metabolomic data underscored their complementary role in characterizing genetic relationships elucidated through DNA fingerprinting techniques. Proteo-metabolomic results verified a certain correlation between the relative abundance of specific polyphenols, enzymes involved in their metabolism, and color characteristics of fruits. These findings highlight the significance of integrating results from diverse *omics* approaches to reveal molecular drivers of ecotype-specific traits and identify biomarkers for selecting and breeding cultivars in the next future.

## 1 Introduction

Sweet cherry (*Prunum avium* L.) is an important fruit species renowned for its sensory appeal and potential health benefits (McCune et al., 2011). The fruits are a valuable source of bioactive compounds, particularly polyphenols, that contribute to their renowned antioxidant and anti-inflammatory properties. Accumulating evidence suggests that sweet cherry consumption may exert protective effects against several chronic diseases, including cardiovascular disorders, diabetes, and neurodegenerative conditions and might exhibit a potential role in the treatment of gout (Blando and Oomah, 2019; Faienza et al., 2020).

Originating from the Caspian and Black Sea regions, sweet cherry cultivation spread across Europe about 2500 years ago, subsequently diversifying into numerous landraces adapted to specific environmental conditions and cultural preferences (Zohary et al., 2012) The past century witnessed the decline of traditional landraces in favor of a few high-performing cultivars selected for economic profitability. Italy, particularly the Campania region, stands as a significant producer of sweet cherries. National and Regional efforts focused on evaluating the genetic diversity of local cultivars to support sustainable agriculture practices and preserve genetic resources (Barreneche et al., 2021; Muccillo et al., 2019).

Phenotypic variations among sweet cherry cultivars encompass fruit characteristics such as size, color, shape, taste, and aroma, as well as traits related to dormancy, flowering, and disease resistance. The development of genetic variability in these ecotypes reflects their adaptation to diverse environmental conditions during their historical spread (Xanthopoulou et al., 2020). Genetic studies employing DNA fingerprinting techniques, such as simple sequence repeat (SSR), random amplification of polymorphic DNA (RAPD), amplified fragment length polymorphism (AFLP), inter simple sequence repeat, single nucleotide polymorphism (SNP) and target region amplification polymorphism (TRAP), contributed to understanding the genetic basis of sweet cherry diversity and evolution (Barreneche et al., 2021; Muccillo et al., 2019). To provide definitive insights into the genetic basis of sweet cherry diversity and evolution, the genomes of key cultivars such as Japanese Satonishiki (Shirasawa et al., 2017), Chinese Tieton (Wang et al., 2020), and Italian Big Star (Pinosio et al., 2020) were independently sequenced, assembled, and annotated. Whole-genome resequencing (WGRS) of multiple sweet cherry accessions representing modern, landrace and wild ecotypes characterized their genetic variations (Pinosio et al., 2020; Shirasawa et al., 2017; Xanthopoulou et al., 2020) identifying millions of SNPs and single nucleotide insertions/deletions. WGRS studies unveiled various domestication and breeding sweeps within the sweet cherry genome, often overlapping to similar regions already identified in *P. persica* (Pinosio et al., 2020). These findings underscore the independent selection pressures acting on similar chromosomal regions/genes during plant domestication processes.

Transcriptomic studies were performed to identify cultivar-dependent molecular differences on two-three accessions having different phenological/phenotypic characteristics (Guo et al., 2018; Wei et al., 2015; Zhai et al., 2022). Concomitant metabolomic (Chen et al., 2022; Michailidis et al., 2020a; Yang et al., 2021) and proteomic (Michailidis et al., 2020a; Xanthopoulou et al., 2022) analyses performed on the same plant tissues of a specific ecotype subjected to transcriptomics punctually assigned metabolic pathways and enzymes affecting different plant physiological processes. These data contributed to elaborate a sweet cherry proteogenomic atlas, in which tissue-specific distribution of quali-quantitative transcriptomic and proteomic information was integrated (Ganopoulou et al., 2021; Xanthopoulou et al., 2022). Advanced metabolomic techniques, including nuclear magnetic resonance (NMR) and liquid chromatography-electrospray ionization tandem mass spectrometry (LC-ESI-MS/MS), were instrumental in characterizing the metabolite profiles of various *P. avium* accessions (Berni et al., 2021; Commisso et al., 2017; Martini et al., 2019) providing insights into intraspecies biodiversity and qualifying cultivars with desirable organoleptic and/or health-promoting features. Two-dimensional (2D) electrophoresis-based proteomics has been used to delineate phenotypic differences between crop ecotypes, to assess the variability within plant populations or to establish hereditary distances for phylogenetic studies (Gomes et al., 2014; Ialicicco et al., 2012; Min et al., 2015; Rossi et al., 2017; Thiellement et al., 1999). Lately, label-free proteomics of selected tissues has been utilized to assess quali-quantitative differences between various pea (Meisrimler et al., 2017), grape (Carpentieri et al., 2019), quinoa (Galindo-Lujan et al., 2021), and narrow-leafed lupin ecotypes (Tahmasian et al., 2022). Notwithstanding their precision but probably depending on their reduced sensitivity due to the requirement of preventive peptide derivatization (Bachor et al., 2019), label-based quantitative proteomics has scarcely been used to evaluate genetic differences, variability, and distance in plant accessions.

Recently, our group has successfully conducted a molecular investigation on the phenotypic diversity of persimmon ecotypes based on the integration of untargeted tandem mass tag (TMT)-based proteomic and metabolomic data (De Pascale et al., 2023). To further assess the suitability of this proteo-metabolomic approach for studying plant genetic diversity, particularly in distinguishing *P. avium* ecotypes at the molecular level, here we have investigated the phenotypic diversity of Italian sweet cherry accessions. These plant ecotypes were chosen based on their diffusion in the Campania Region and the corresponding commercial distribution and value. In this context, Italy is among the top-five cherry producers countries worldwide (https://www.tridge.com/intelligences/sweet-cherry/IT/production), and the Campania Region is the second national district for fruit production quantities. Bioinformatic analysis of resulting proteo-metabolomic data yielded coherent insights into the phenotypic relationships of these cultivars. This initial comparison of fruit cultivars at the relative molecular composition level will facilitate the development of dedicated targeted quantitative proteomic and/or metabolomic procedures to assess fruit composition standards for further breeding and quality control activities, thus addressing consumer and market demands.

## 2 Materials and methods

### 2.1 Chemicals

Methanol, acetonitrile and water of mass spectrometry-grade quality were purchased from Merck Sigma-Aldrich (Darmstadt, Germany). Analytical-grade potassium persulfate, sodium chloride, formic acid, sodium dihydrogen phosphate dihydrate, di-sodium hydrogen phosphate dihydrate, as well as Trolox and Folin-Ciocalteu′s reagents were obtained from Merck Sigma-Aldrich. All the other chemicals were of analytical grade and were purchased from Merck Sigma-Aldrich, unless otherwise specified.

### 2.2 Plant material and morphological evaluation

The study focused on various sweet cherry genotypes from the Campania region, namely Della Recca (DRecc), Della Signora (DSig), Del Monte (DMon), Pagliaccio (Pagl), Palermitana (Pal), Palermitana Terzaiola (PalT), Pellicciara (Pell), Tamburella (Tamb), Cannamela (Cann) and Imperatore (Imp), sourced from the same *ex situ* germplasm collection situated at the experimental farm of the Center for Applied Agricultural Research in Eboli (Salerno), Italy (40°33’32”N, 14°58’09”E). As result of concomitant tree planting for agrobiodiversity conservation activities, sweet cherry trees of each genotype were the same age. Morphological evaluations were conducted based on forty-one descriptors outlined by the International Union for the Protection of New Varieties (TG_35_7; UPOV, 2006), as detailed in **Supplementary Table S1**. Sweet cherry fruits from each genotype (n=130) were hand-harvested at the commercial ripening stage (BBCH 89), as defined according to the Biologische Bundesanstalt, Bundessortenamt, and CHemische Industrie (BBCH) scale; the above-reported stage value corresponds to full fruit ripeness. Furthermore, for each genotype, skin color and total soluble solids content (SSC) were evaluated. The SSC value of each cultivar was always greater than 10 Brix, which was also suggestive of full fruit ripeness. Fruit samples were harvested from three trees of each genotype, ensuring the absence of qualitative/mechanical defects; fruit sampling was performed at the four cardinal points of each tree. Harvested fruits of each cultivar were randomly divided into three pooled biological samples that were then subjected to different analyses.

### 2.3 Physicochemical characterization of fruits

Total SCC and titratable acidity (TA) values of each cultivar were determined from fresh juice extracted from 20 fruits using an electric juice extractor. SSC (°Brix) and TA (malic acid g/L) were quantified using a DBR35 digital refractometer (Sinergica Soluzioni, Pescara, Italy) and by titration with 0.1 M NaOH, respectively. The skin overcolor was assessed at two equatorial points of 20 fruits of each cultivar using chromaticity values L* (lightness), a* (green to red), and b* (blue to yellow) measured with a CR5 colorimeter (Minolta Camera Co., Japan). Total polyphenol concentration (POL) and antioxidant activity (AOX) of 20 fruits of each cultivar were determined using the Folin–Ciocalteu method and the 2-diphenyl-2-picryl-hydrazil (DPPH) method, respectively (Goffi et al., 2020). Results are expressed as µmol of gallic acid equivalents (GAE) per g of fresh weight (FW) and as µmol Trolox equivalent (TE) per g of FW, respectively. Total protein (TP) content of 10 fruits of each cultivar was determined using the Kjeldahl method following ISO 1871:2009 guidelines (ISO, 2009). After sample mineralization with a Foss Tecator™ 2508 device (Fisher Scientific, USA), total nitrogen content was measured with a Foss Kjeltec™ 8200 instrument (Fisher Scientific, USA). All physicochemical data were assessed on three biological replicates of each cultivar; they are presented as mean values ± standard deviation and were analyzed by one-way ANOVA. Mean values were compared using Tukey’s test with a significance level of α = 0.05 employing SPSS software package, v. 20.0 (SPSS, Chicago, IL, USA).

### 2.4 Proteomic analysis

#### 2.4.1 Protein extraction

Fruits (n=60) from each cultivar were pooled in three biological replicates and separately pulverized in liquid N_2_ with a pestle and mortar, and finally lyophilized. Proteins were extracted in parallel using a modified version of a phenol-based extraction protocol (De Pascale et al., 2023). Specifically, 1 g of fine powder, supplemented with 1% of polyvinylpyrrolidone, was mixed in a resuspension buffer consisting of 0.7 M sucrose, 0.1 M KCl, 0.5 M Tris-HCl (pH 8.8), 50 mM EDTA, 40 mM DL-dithiothreitol, 1 mM phenylmethylsulfonyl fluoride, and the protease inhibitors cocktail for plant tissues (Sigma-Aldrich, USA) (0.2 mL of inhibitor solution in 10 ml cell lysate from about 6 g of fresh plant tissue). The mixture was homogenized on ice using a T10 basic Ultra-Turrax™ (IKA, Staufen, Germany) with 5 cycles of 10 s at 20,000 rpm, interspersed with 20-s pauses. An equal volume of saturated phenol (Sigma-Aldrich) in 10 mM Tris-HCl, pH 8.1 was added, and the mixture was shaken for 20 min, at 4°C. Subsequently, the biological samples of each cultivar were centrifuged at 10,000 x *g* for 10 min, at 4°C, and the corresponding phenolic phase was independently collected. Each phenolic phase was subjected to back-extraction with 3 mL of resuspension buffer, vortexed for 3 min, and recovered by centrifugation at 10,000 x *g* for 10 min, at 4°C. To precipitate proteins, six vol of ice-cold 0.1 M ammonium acetate in methanol were added and the mixture was left overnight, at -20°C. After centrifugation (10000 x *g*, 10 min, 4°C), the pellets were washed three times with ice-cold 0.1 M ammonium acetate in methanol, and three times with ice-cold acetone. The protein pellets were then air-dried and solubilized in 600 µL of an aqueous solution containing 8 M urea, 50 mM triethylammonium bicarbonate (TEAB), pH 8.5. Samples were vortexed, sonicated in ultrasonic bath for 5 min, and shook at 22°C, overnight. After centrifugation at 16000 x *g* for 30 min, at 22°C, protein concentration was determined on each supernatant using the Pierce BCA Protein Assay kit™ (Thermo Scientific, Rockford, IL), following the manufacturer’s instructions. The quality of protein extracts was finally assessed through SDS-PAGE, which was performed with a SE600 Chroma™ device (Hoefer, Holliston, MA) and followed by staining with Coomassie Brilliant Blue G-250 (Bio-Rad, Hercules, CA) (data not shown).

#### 2.4.2 Protein identification and relative quantitation

Quantitative proteomics of sweet cherry fruits was conducted using the tandem mass tag (TMT) approach, as already described for other plant tissues (De Pascale et al., 2023; Lombardi et al., 2020; Samperna et al., 2021). Briefly, 100 µg of proteins for each cultivar, as deriving from the combination of the three biological replicates reported above, were diluted to a final volume of 100 µL with 100 mM TEAB, and then reduced with 5 µl of 200 mM tris(2-carboxyethylphosphine), for 60 min, at 55°C. Subsequently, protein samples were separately treated with 5 µL of 375 mM iodoacetamide in the dark, for 30 min, at 25°C. Alkylated proteins were precipitated in parallel by addition of 6 vol of ice-cold acetone at -20°C, overnight, followed by centrifugation at 8,000 *g* for 10 min, at 4°C. Then, the protein pellets were air-dried, parallelly solved in 100 mM TEAB and trypsinolyzed. Trypsin was added to each protein sample at a 1:50 trypsin-to-protein mass ratio for overnight digestion at 37°C. The resulting peptides from each protein sample were labelled with the TMT10plex Label Reagent Set (Thermo-Fisher Scientific, USA) according to the manufacturer’s protocol. Each sample was labeled as follows: TMT^10^-126: PalT; TMT^10^-127N: Pal; TMT^10^-127C: Tamb; TMT^10^-128N: Pagl; TMT^10^-128C: Pell; TMT^10^-129N: Imp; TMT^10^-129C: DRecc; TMT^10^-130N: Cann; TMT^10^-130C: DMon; TMT^10^-131: DSig. Following labeling (1 h), each reaction was quenched by adding 8 µL of 5% w/v hydroxylamine, and mixed for 15 min. Tagged peptide mixtures were then combined in equal-molar ratios (1:1:1:1:1:1:1:1:1:1), vacuum-dried with a rotoevaporator (SpeedVac, Thermo Fisher Scientific, Bremen, Germany), and finally fractionated with the Pierce™ High pH Reversed-Phase Peptide Fractionation kit (Thermo Fisher Scientific) following the manufacturer’s instructions. Eight fractions of TMT-labelled peptides were collected, vacuum-dried, and reconstituted in 0.1% formic acid.

Peptide mixtures were analyzed in technical triplicate through a nanoLC-ESI-Q-Orbitrap-MS/MS apparatus consisting of an UltiMate 3000 HPLC RSLCnano system holding an Acclaim PepMapTM RSLC C18 column (150 mm ×75 mm ID, 2 mm particles, 100 Å pore size), which was connected to a Q-Exactive Plus mass spectrometer equipped with a Nanoflex ion source (all manufactured by Thermo Fisher Scientific). Peptides were eluted with water/acetonitrile/formic acid 19.92/80/0.08 v/v/v (solvent B) and water/formic acid 99.9/0.1 v/v (solvent A), running at an overall flow rate of 300 nL/min. The gradient started with an isocratic phase of 20 min at 5% of solvent B, followed by a linear increase to 60% over 125 min, a ramp to 95% over 1 min, maintenance at 95% for 8 min, and finally return to the initial condition in 1 min. The mass spectrometer operated in data-dependent mode, employing a full MS scan (*m/z* range 375–1500) at nominal resolution of 70,000 (at m/z 200), followed by MS/MS scans of the 10 most abundant ions. MS/MS spectra were acquired in the *m/z* range 110–2000, with a normalized collision energy of 32%, an automatic gain control target of 100,000, a maximum ion target of 120 ms, and a resolution of 17,500. A dynamic exclusion value of 30 s was applied.

#### 2.4.3 Bioinformatic analysis of proteomic data

Mass spectrometric data were processed for protein identification and relative quantification using Proteome Discoverer v. 2.4 (PD2.4) software (Thermo Scientific), and database search by the Mascot algorithm v. 2.6.1 (Matrix Science, UK) against the *P. avium* UniProtKB protein database (31,684 protein sequences), integrated with most common protein contaminants. The data search criteria included TMT10-plex modification of Lys and peptide *N*-terminus, and Cys carbamidomethylation as fixed modifications, as well as Asn/Gln deamidation, and Gln/Glu pyroglutamate formation as variable modifications. Peptide and fragment mass tolerances were set to ± 10 ppm and ± 0.05 Da, respectively. Trypsin was selected as the proteolytic enzyme, with a maximum of 2 missed cleavages allowed. Confident protein identification required at least two sequenced peptides and an individual peptide Mascot Score ≥ 30, with results filtered to 1% false discovery rate. Protein abundance values were derived from the intensities of TMT reporter ions. Statistical analysis was performed using PD2.4. A one-way ANOVA test with TukeyHSD *post-hoc* test was applied to the abundance of individual proteins across the ten cultivars to identify statistically significant differences. The test yielded p-values associated with the abundance ratios between single cultivars. A p-value < 0.05 was considered statistically significant. The differentially represented proteins (DRPs) were selected if they significantly changed in at least one by one comparison between cultivars (p-value ≤ 0.05) showing an abundance fold change (FC) corresponding to log_2_FC ≥ 1.0 or log_2_FC≤ -1. Hierarchical clustering analysis of DRPs was performed by Proteome Discoverer 2.4 (De Pascale et al., 2023) on normalized abundance values (total peptide amount) employing the “scale before clustering” function, with Pearson distance and average linkage method as clustering metrics. The proteomic data were deposited in the ProteomeXchange Consortium via the PRIDE partner repository (Perez-Riverol et al., 2022) with the dataset identifier PXD051478.

Functional analysis was performed as previously reported (Salzano et al., 2019), performing a preliminary classification with Mercator4 v5.0 software (Lohse et al., 2014) followed by automatic Kyoto Encyclopedia of Genes and Genomes (KEGG) mapping and KEGG Orthology (KO) assignment through Blast KEGG Orthology and Links Annotation (KOALA) web-service (Kanehisa et al., 2016). Functional enrichment analysis of the DRPs was obtained for biological processes (Gene Ontology) and KEGG pathways through STRING software (https://string-db.org). Statistical analyses of protein abundance data were conducted using GraphPad Prism 6.0 (GraphPad Software, La Jolla, CA, USA), or XLStat (v. 5.03, Addinsoft, NY, USA).

### 2.5 Metebolomic analysis

Polyphenols were extracted following a dedicated procedure developed in our group for vegetable products, including fruits, berries, leaves, and drupes (De Pascale et al., 2023). Freeze-dried cherries aliquoted in three biological replicate pools for each cultivar were pulverized by using a knife-mill Grindomix GM200 (Retsch, Haan, Germany), and 0.2 mg of the resulting powder was mixed with 1 mL of hydroalcoholic solution (methanol/water, 70:30 v/v). The suspensions were vortexed for 5 min at 1000 rpm and then sonicated in an ice bath for 15 min. Fruit extract samples of each cultivar were independently centrifuged (2600 x *g*), and supernatants were diluted ten times in water/methanol/formic acid 84.9:15:0.1 v/v for further liquid chromatography-electrospray ionization- tandem mass spectrometry (LC-ESI-MS/MS) analysis. Data were acquired using an LTQ Orbitrap XL hybrid Fourier transform mass spectrometer interfaced with an ultra-high performance liquid chromatography system (Ultimate 3000 RS, Thermo Fisher Scientific). Separation of phytochemicals was achieved in reversed-phase mode using a Kinetex PS C18 column (100 x 2.1 mm, 2.6 µm ID, Phenomenex), thermostated at 35°C. The acid mobile phases enhanced the response of the flavylium ion signals and consisted of 1% formic acid in water (solvent A) and 1% formic acid in methanol (solvent B). Samples (5 µL) were injected in full loop mode and analytes were resolved using a gradient of solvent B (minutes/%B): (0/10), (2/10), (12.5/55), (14/55), (17/95), (19/95), at a flow rate of 0.2 mL/min, with an equilibration stage of 5 min at 10% of mobile phase B. Analytes were alternatively detected in positive and negative ion modes in a top three untargeted data-dependent scanning mode. ESI interface spray voltage and capillary voltage were 4.8 kV and +38 V for positive ions, and -4.2 kV and -70.0 V for negative ions, respectively. Capillary temperature was 275°C; sheath gas and auxiliary gas flow values were 25 and 3 arbitrary units, in both modalities. Profile data were acquired in full scan Fourier transformed mass spectrometry (FTMS) mode with a mass scanning range of *m/z* 70–1200 and a resolution of 30000 (FWHM at *m/z* 200). For associated data-dependent scanning experiments, MS/MS normalized collision energy was set to 25, activation Q to 0.25, activation time to 25 ms, with a 1 *m/z* isolation window and a resolution of 7500. A reject mass list was generated by injecting blank samples.

An *in-house* mass list was compiled in Xcalibur v. 2.1 and Trace Finder v. 5.1(Thermo Fisher Scientific) using available anthocyanins and anthocyanidins from Phenol-explorer (www.phenol-explorer.eu) and FooDB (www.foodb.ca). This dataset was supplemented with polyphenol chemical structures available in other public databases such as mzCloud (www.mzcloud.org), PhytoHub (www.phytohub.eu) and MassBank (www.massbank.eu). Tandem mass spectra were manually curated to assign annotations based on reproducibility of analyte retention time, chemical formula, exact mass, isotopic pattern and MS/MS profiles. Signal correction and normalization were achieved through quality control samples obtained by mixing hydroalcoholic supernatants of each sample in equal amount. Area counts of target analytes were subjected to multivariate data analysis using XLStat (v. 5.03, Addinsoft, NY, USA). A heat-map from red to blue through white was optimized using centering and reduction procedures to enhance associations between cultivars (dendrograms on the x axis) and analytes (dendrograms on the y axis) following Euclidean distance and the Ward linkage method. Linear correlation analysis between the abundance values of 63 DRPs and 64 metabolites was performed with OriginPro 2023 software (OriginLab, Northampton, MA), using Pearson as the correlation type. Correlations with *p-value* < =0.01 were considered statistically significant.

## 3 Results

### 3.1 Physicochemical characteristics of sweet cherry fruits

The phenological and agronomic traits of sweet cherry DRecc, DSig, DMon, Pagl, Pal, PalT, Pell, Tamb, Cann and Imp cultivars from the Campania region were determined and defined according to the descriptors of the International Union for the Protection of New Varieties of Plants (UPOV) (**Supplementary Table S1**). Fruits with a homogeneous allocation over the trees and the same maturation degree were harvested and further characterized by their qualitative traits. Cherries from different accessions exhibited distinct physical attributes concerning skin, flesh, stone, and size (**Supplementary Figure S1**). The recorded average fruit weight spanned from a minimum of 4.23 ± 0.76 g for the Pell ecotype to a maximum of 8.76 ± 1.34 g for the Pagl accession (**Table 1**), aligning with previous findings (Di Matteo et al., 2017). As expected, evident differences regarding colorimetric coordinates (L*, a* and b*) were observed between ecotypes (**Table 1**).

**Table 1:** Pomological and fruit qualitative traits of Della Recca (DRecc), Della Signora (DSig), Del Monte (DMon), Pagliaccio (Pagl), Palermitana (Pal), Palermitana Terzaiola (PalT), Pellicciara (Pell), Tamburella (Tamb), Cannamela (Cann) and Imperatore (Imp) sweet cherry cultivars.

Cultivar	Fruit Weight (g)	L*	a*	b*	SSC (°Brix)	TA (mg malic acid/L)	pH	TPC (mg GAE/ 100g FW)	AOX (µmol Trolox/ g FW)	TP (g/100 g FW)
Cann	5.30 ± 0.95^b^	37.04 ± 5.78^d^	52.56 ± 3.18^d^	36.39 ± 4.63^d^	15.27 ± 0.64^bc^	7.47 ± 0.28^b^	3.63 ± 0.01^ab^	280.11 ± 10.28^e^	4.92 ± 0.17^d^	0.94 ± 0.02
DSig	4.60 ± 0.64^ab^	47.56 ± 9.79^e^	53.22 ± 11.11^d^	42.81 ± 9.31^e^	17.07 ± 1.70^de^	12.72 ± 0.40^d^	3.49 ± 0.12^a^	66.33 ± 4.08^a^	2.27 ± 0.00^a^	1.24 ± 0.02
DRecc	4.84 ± 0.51^ab^	43.30 ± 4.06^e^	53.73 ± 2.02^d^	37.80 ± 2.09^d^	16.07 ± 0.25^cd^	10.17 ± 0.49^c^	3.89 ± 0.09^bcd^	160.50 ± 7.08^c^	4.86 ± 0.09^cd^	1.13 ± 0.02
DMon	7.77 ± 0.72^d^	9.87 ± 2.67^a^	21.25 ± 5.41^ab^	7.98 ± 4.40^ab^	19.80 ± 0.60^f^	14.00 ± 0.40^e^	3.39 ± 0.35^a^	273.92 ± 17.03^e^	5.45 ± 0.06^e^	0.84 ± 0.02
Imp	6.40 ± 0.97^c^	23.71 ± 5.13^c^	38.17 ± 8.72^c^	22.99 ± 8.94^c^	16.20 ± 1.74^cde^	8.05 ± 0.51^b^	3.44 ± 0.05^a^	246.48 ± 9.32^d^	4.76 ± 0.11^cd^	1.22 ± 0.02
Pagl	8.76 ± 1.34^e^	11.73 ± 5.06^a^	16.38 ± 3.28^a^	4.11 ± 2.45^a^	17.93 ± 1.10^e^	12.00 ± 1.33^d^	3.68 ± 0.34^abc^	174.43 ± 8.35^c^	4.67 ± 0.25^c^	1.09 ± 0.02
Pell	4.23 ± 0.76^a^	38.23 ± 7.67^d^	51.64 ± 2.21^d^	39.55 ± 1.52^de^	17.95 ± 0.60^e^	12.70 ± 0.70^d^	3.39 ± 0.07^a^	126.69 ± 14.69^b^	4.76 ± 0.03^cd^	1.07 ± 0.02
Pal	4.77 ± 0.52^ab^	18.86 ± 2.67^b^	26.27 ± 3.79^b^	11.36 ± 2.89^b^	11.77 ± 0.55^a^	5.10 ± 0.80^a^	4.00 ± 0.08^cd^	133.96 ± 13.80^b^	4.90 ± 0.09^d^	1.04 ± 0.02
PalT	4.42 ± 0.55^a^	18.63 ± 2.22^b^	22.04 ± 4.40^b^	7.74 ± 4.17^ab^	13.80 ± 0.30^b^	4.80 ± 0.40^a^	4.14 ± 0.25^d^	129.11 ± 14.53^b^	3.81 ± 0.10^b^	1.09 ± 0.02
Tamb	6.75 ± 0.55^c^	13.61 ± 3.52^a^	22.26 ± 5.26^b^	8.23 ± 4.15^ab^	13.60 ± 0.40^b^	5.40 ± 0.60^a^	3.86 ± 0.09^bcd^	250.24 ± 23.44^d^	6.22 ± 0.06^f^	0.84 ± 0.02

Reported are data on fruit fresh weight (FW), colorimetric coordinates (L*, a* and b*), soluble sugar content (SSC), titratable acidity (TA), pH, total polyphenol content (TPC), antioxidant activity (AOX), and total protein content (TP). Reported values correspond to the mean ± SD. GAE, gallic acid equivalents; FW, fresh weight. Data are reported as mean values ± standard deviation. Differences among cultivars within each column were analyzed by ANOVA with a confidence interval of 95%. Different letters indicate a significant difference (Tukey test, P < 0.05).

Total soluble solids content (SSC) exhibited significant differences among cultivars, ranging from 11.77± 0.55 (Pal) to 19.80 ± 0.60° Brix (DMon) (**Table 1**). On the other hand, titratable acidity (TA) manifested the lowest values in Pal, PalT and Tamb accessions, and the highest value in the DMon ecotype (**Table 1**). Polyphenols are natural antioxidant compounds imparting both flavor and potential health-promoting characteristics to sweet cherries (Clodoveo et al., 2023). Notably, the DSig cultivar simultaneously exhibited the lowest total polyphenol content (TPC) and antioxidant activity (AOX) values compared to all sweet cherry ecotypes (**Table 1**). Data closely resembled those reported in previous studies (Mirto et al., 2018; Pacifico et al., 2014), although showing discrepancies in some cases (Di Matteo et al., 2017). Regarding total protein content (TP), no major differences were observed between accessions (**Table 1**).

### 3.2 Comparative proteomic analysis of sweet cherry accessions

**Figure 1:**
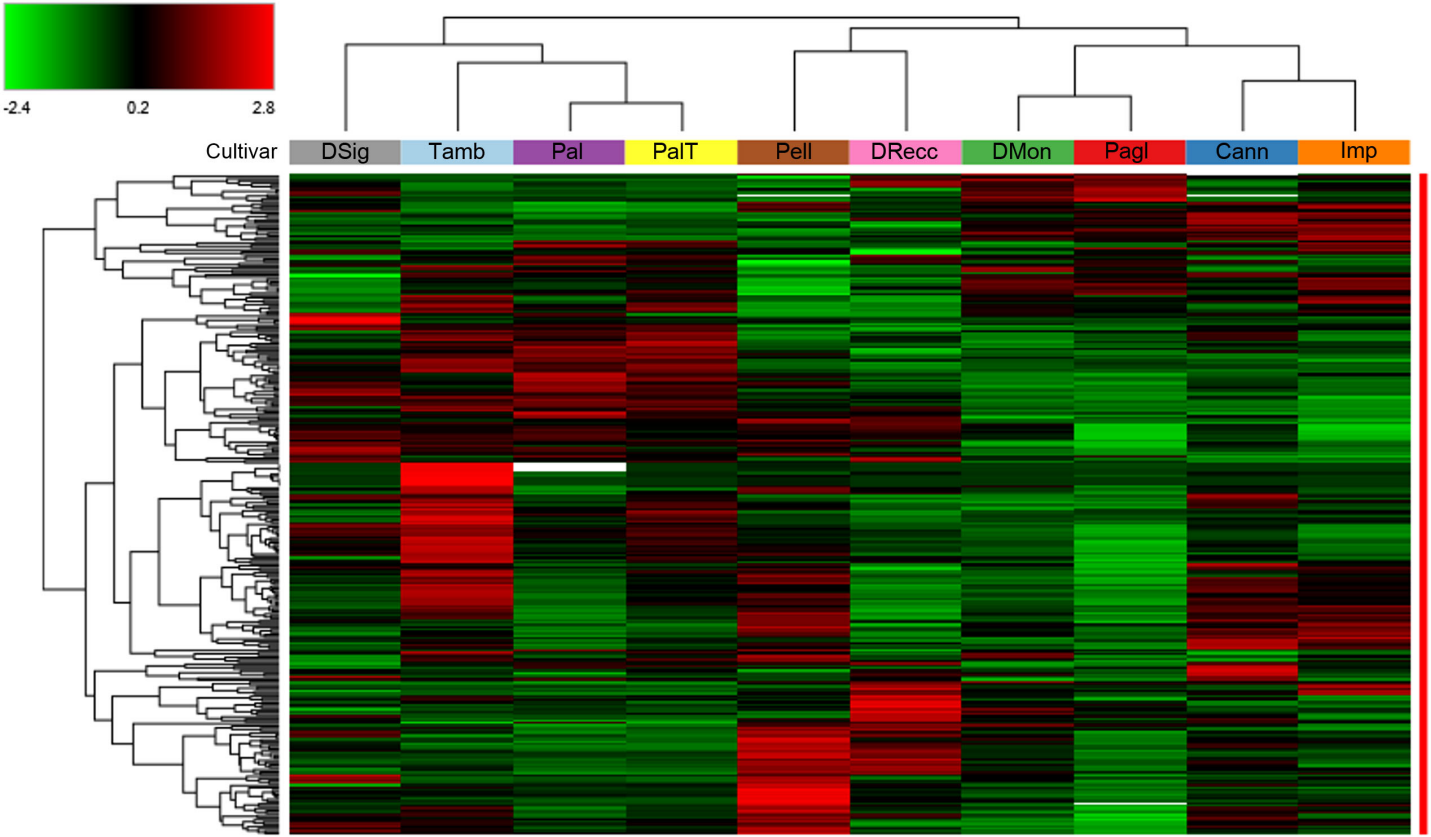
Hierarchical clustering of the 288 differentially represented proteins (DRPs) identified in the TMT-based proteomic analysis of ten sweet cherry cultivars. The heatmap displays normalized (total abundance normalization) and scaled protein abundance values, with color intensity indicating relatively higher (red) and lower (green) abundance levels. Hierarchical clustering was performed using Pearson distance and the average linkage method. Rows represent DRPs (*p* < 0.05, log_2_FC≥1 and log_2_FC≤ -1), and columns correspond to sweet cherry cultivars. Plant cultivars with similar proteomic profiles were effectively grouped based on the similarity of their protein expression patterns. Detailed information on DRPs shown in this figure is provided in **Supplementary Table S2**.

A comprehensive proteomic description of the above-reported cherry cultivars was achieved through a differential experiment using TMT-labeling of protein digests and nanoLC-ESI-Q-Orbitrap-MS/MS analysis. This approach led to the identification of 3786 proteins from the UniProtKB database sequences specific to the *P. avium* species, adhering to the established acceptance criteria outlined in the method section. A subset of 288 differentially represented proteins (DRPs) was extracted by filtering pairwise abundance ratios data, according to the criteria of p-value ≤ 0.05, log_2_FC≥1 or log_2_FC≤ -1 (**Supplementary Table S2**). These proteins were subsequently used for cultivar discrimination. A heat map representation of protein abundances derived from the 288 DRPs revealed the presence of four clusters corresponding to specific sweet cherry cultivars (**Figure 1**).

**Figure 2:**
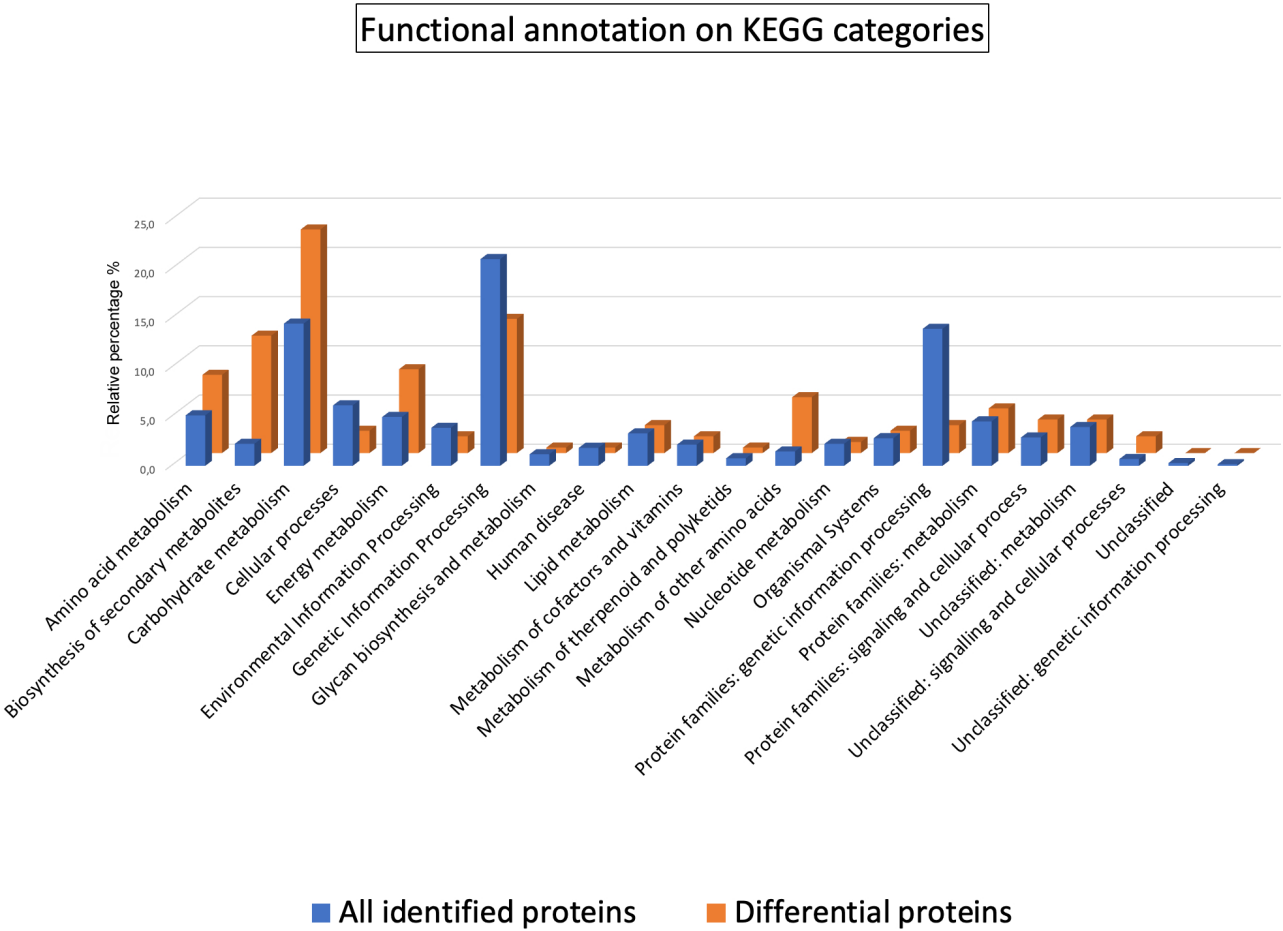
Functional classification of the proteins identified and quantified in the sweet cherry cultivars. Histograms represent the relative percentages of KO functional categories within the annotated sets of all identified proteins (blue) and the subset of DRPs (orange).

To unravel molecular disparities between the sweet cherry cultivars, a functional assignment of the 288 DRPs was conducted using Mercator4 software (**Supplementary Figure S2**). As expected, Enzymes and Unassigned proteins, being general categories, were the most represented (27% and 23%, respectively). Interestingly, Secondary metabolism (6%) emerged as the most represented specific category, suggesting potential variations in the biosynthesis of secondary metabolites among the ecotypes. An automatic annotation through the KEGG Ontology assignment was further executed to discern metabolic pathways potentially contributing to observed cultivar differences. KO analysis showed that 60.9% and 64.9% of the protein entries were annotated in the 288 DRPs and all identified proteins, respectively. This analysis facilitated the assignment of individual gene functions and the reconstruction of the corresponding KEGG pathways. The distribution of KO functional categories for the entire set of identified proteins and the DRPs subset has been illustrated in **Figure 2**.

The comparative analysis of KO categories revealed a marked enrichment in the DPRs subset of several classes with respect to all identified proteins as follows: Biosynthesis of other secondary metabolites (11.9% vs. 2.2%), Metabolism of other amino acids (5.7% vs. 1.5%), Energy metabolism (8.5% vs. 5.0%), Amino acid metabolism (8.0% vs. 5.1%), Carbohydrate metabolism (22.7% vs. 14.5%). Specifically, 63 proteins assigned to 50 KO identifiers were encompassed within the category of Biosynthesis of secondary metabolites (k01110) (**Supplementary Table S3**) and primarily associated with flavonoid biosynthetic processes (**Supplementary Figure S3**).

**Figure 3:**
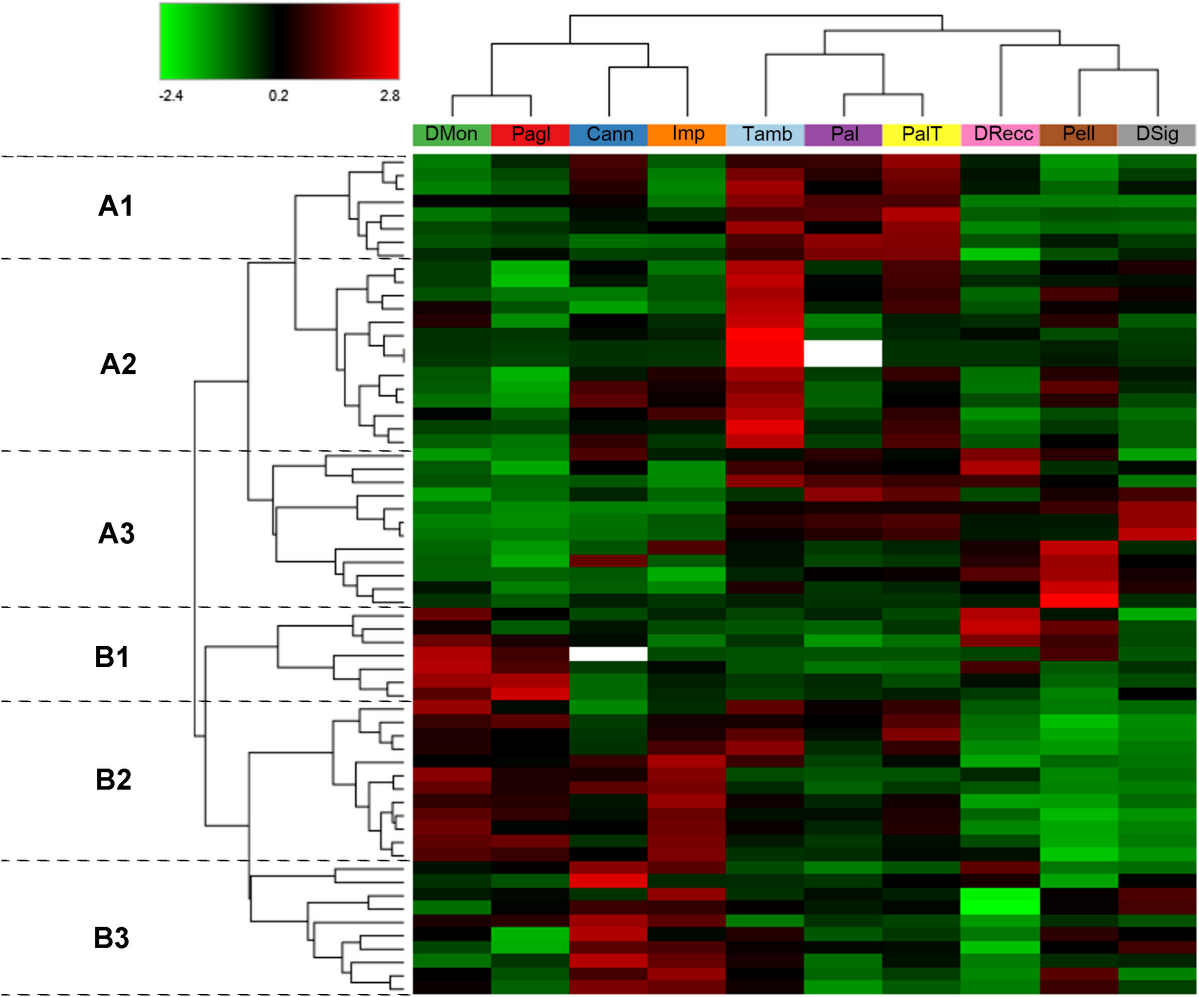
Hierarchical clustering of the 63 differentially represented proteins (DRPs) classified under the KEGG Orthology category “Biosynthesis of secondary metabolites” (k01110) in sweet cherry cultivars. The heatmap displays normalized (total abundance normalization) and scaled protein abundance values, with color intensity indicating relatively higher (red) and lower (green) abundance levels. Hierarchical clustering was performed using Pearson distance and the average linkage method. Rows represent DRPs (*p* < 0.05, log_2_FC≥1 and log_2_FC≤ -1) involved in the biosynthesis of secondary metabolites, and columns correspond to sweet cherry cultivars. Two main protein clusters **(A, B)** were identified, each subdivided into three sub-clusters **(A1-A3, B1-B3)**. Plant cultivars with similar protein abundance profiles are grouped together. Detailed information on the DRPs shown in this figure is provided in **Supplementary Table S3**.

Therefore, a heatmap and hierarchical clustering of these DRPs were generated to visualize the differences in protein abundance among individual accessions (**Figure 3**). Cultivars were stratified into three primary groups based on protein abundance profiles as follows: DMon, Pagl, Cann and Imp (group 1); Tamb, Pal and PalT (group 2); DRecc, Pell and DSig (group 3). Of note, this clustering scheme bears strong similarities to that obtained on the entire set of 288 DRPs (**Figure 1**), with the main difference uniquely concerning the gathering of the cultivar DSig.

The cultivars grouped under group 1 exhibited notable differentiation from those in group 2 and group 3, particularly concerning the proteins in the cluster B2 (**Figure 3**). These proteins are primarily involved in the flavonoid biosynthetic process (*e.g.*, cinnamate 4-hydroxylase, C4H; F chalcone synthase, FLS; chalcone synthase, CHS; chalcone isomerase, CHI; flavonoid 3-hydroxylase, F3H; anthocyanidin synthase, ANS; dihydroflavonol 4-reductase, DFR; flavonoid 3’-hydroxylase, F3’H; UDP-glycosyltransferase 88F5-like, UFGT), and anthocyanin biosynthesis (anthocyanidin 3-O-glucosyltransferase, BZ1). These proteins were more prominently represented in the accessions of group 1 (DMon, Pagl, Cann, and Imp), while they were less abundant in the ecotypes of group 3 (DRecc, Pell, DSig), with intermediate abundance observed in the cultivars of group 2 (Tamb, Pal and PalT), (**Figure 3**). Within group 1, DMon and Pagl cultivars were separated from Cann and Imp accessions due to the higher representation of proteins in cluster B1. This cluster encompasses proteins related to glycolysis (*e.g*., glyceraldehyde-3-phosphate dehydrogenase, GAPDH; phosphoglycerate kinase, PGK; phosphoenolpyruvate carboxykinase), biosynthesis/metabolism of amino acids (*e.g.*, catalase, (R)-mandelonitrile beta-glucosyltransferase, UGT85A19; shikimate dehydrogenase) and coniferyl-aldehyde dehydrogenase, REF1. Moreover, Cann and Imp ecotypes were characterized by a high abundance of proteins in cluster B3, primarily involved in the phenylpropanoid biosynthetic process (*e.g*., alcohol dehydrogenase class-P; peroxidase) and biosynthesis of amino acids (*e.g.*, alcohol dehydrogenase, ADH and ADH1; catalase; S-adenosylmethionine synthase; aspartokinase, thrA; alpha-amylase; N-acyl-L-amino-acid amidohydrolase).

The cultivars belonging to group 2 (Tamb, Pal and PalT) displayed key differences from other ecotypes primarily due to the prevalence of proteins in cluster A1, including Rubisco, small and large subunits (rbcS and rbcL), several enzymes involved in phenylpropanoid biosynthesis (*e.g*., caffeoyl-CoA-O-methyltransferase; peroxidase; phenylalanine ammonia-lyase, PAL), 12-oxophytodienoate reductase 2-like implicated in jasmonic acid biosynthesis, and primary amine oxidase participating in amino acid metabolism. Additionally, the Tamb ecotype exhibited a notably high abundance of proteins in cluster A2, encompassing enzymes involved in amino acid metabolism [(R)-mandelonitrile lyase, MDL3; shikimate dehydrogenase, aroDE; bifunctional aspartate aminotransferase and glutamate/aspartate-prephenate aminotransferase, PAT; fructose-bisphosphate aldolase; glutamate decarboxylase], phenylpropanoid biosynthesis (*e.g*., cytochrome P450 98A2-like, CYP98A; caffeic acid 3-O-methyltransferase COMT; probable cinnamyl alcohol dehydrogenase 1, CAD; phenylalanine ammonia-lyase, PAL), carotenoid biosynthesis (*e.g*., 9-cis-epoxycarotenoid dioxygenase, NCED1), fatty acid biosynthesis (acetyl-CoA carboxylase), linoleic acid metabolism (*e.g.*, lipoxygenase, LOX2S), and carbohydrate metabolism (sucrose synthase, SUS).

### 3.3 Comparative analysis of allergens in sweet cherry accessions

Allergy to sweet cherries is often reported to be associated with allergic reactions to other fruits within the Rosaceae family and tree pollinosis due to cross-reactive allergens (Fuchs et al., 2006). By using a TMT-based proteomic approach, we identified several sequence entries corresponding to allergenic *P. avium* proteins reported in the Allergome database (www.allergome.org). Among that, we identified multiple isoforms of the following allergens: Pru av 1 (Bet v 1-like protein), recognized as the major allergen of sweet cherry fruit (Ippoushi et al., 2016), Pru av 2 (thaumatin-like protein), Pru av 3 (non-specific lipid-transfer protein) and Pru av 4 (profilin) (**Supplementary Table S4**). We did not detect Pru av 7 (gibberellin), which has been recently acknowledged as an emerging sweet cherry allergenic component (Iizuka et al., 2022). For a quantitative assessment of the relative abundances of the above-mentioned allergens among different cultivars, we focused on the most abundant isoform of each molecular species (**Supplementary Table S4**), and the outcomes are reported in **Supplementary Figure S4A**. The quantitative trend of allergen abundances across the cultivars was similar for Pru av2, Pru av3 and Pru av4. Conversely, a distinct tendency was observed for Pru av 1, which showed a very low relative allergen content in the cultivar Pell and high relative protein levels in Pagl, DRecc, DSig and DMon accessions. When considering the cumulative contribution of all allergenic proteins, it was evident that the ecotypes Pell and Pagl exhibited the lowest and the highest relative content of allergens, respectively, primarily due to the abundance of Pru av1 (**Supplementary Figure S4B**). Absolute quantification of the different allergens would necessitate dedicated methods, generally relying on the combined use of isotopically labelled peptides and LC-ESI-MS/MS, similarly to what was already accomplished for the determination of Pru av 2 in various fruit types (frozen and dried) or processed products (jelly and jam) (Ippoushi et al., 2016).

### 3.4 Comparative analysis of phytochemicals in sweet cherry accessions

To accomplish a qualified description of cherry cultivars as referred to their polyphenol levels, we used an analytical workflow based on a reversed-phase core-shell stationary phase able to interact with aromatic rings of analytes in their aglycone or glycosidic forms and promote their efficient separation. Given the presence of positively charged substituents at pH below 3, we optimized the separation of flavyilium ions characteristic of anthocyanins and their aglycons through their hydrogen bonding accepting capacity and steric interaction, using a dedicated 19-min LC gradient. For mass spectrometric identification of analytes, we constructed an in-house mass list based on the chemical nature of anthocyanins, the pivotal polyphenol biomarkers of cherries. In accordance with the methodology described by Troise and coworkers (Troise et al., 2014), we compiled all the putative chemical compounds in a dedicated mass list and subsequently developed an untargeted mass spectrometry-based protocol in data-dependent scanning mode within a pseudo-targeted identification procedure. This approach outlined the different types of secondary metabolites synthesized by specific enzyme classes already ascertained through proteomics, as recently described by our group for persimmon (De Pascale et al., 2023). **Supplementary Table S5** provides details on the analytical performances of the identified analytes belonging to various polyphenol classes as hydroxycinnamic acids, hydroxybenzoic acids, flavanols, flavones, flavonols, flavanones (all detected in negative ion mode, [M-H]^-^) and anthocyanidins (solely detected in positive ion mode [M]^+^) (Amarowicz et al., 2009).

In both positive and negative ion modes, the detection of theoretical mass values in full scan mode included a mass accuracy within ± 5 ppm. Among the hydroxycinnamic acids, cherry samples exhibited the presence of derivatives of caffeic acid, ferulic acid, and coumaric acid with diagnostic ions at *m/z* 135, 193 and 191, respectively. Among hydroxybenzoic acids, protocatechuic acid was the only aglycone detected (153.01933 → 109), while the other three compounds were present in their glycosidic form, namely vanillic acid 4-*O*-glucoside, 4-hydroxybenzoic acid 4-*O*-glucoside, protocatechuic acid 4-*O*-glucoside.

The flavanols sub-class included catechin and epicatechin (retention times 7.2 and 7.9 min, respectively) along with procyanidin dimers and trimers as well as epicatechin 3-*O*-gallate (Picariello et al., 2016). Luteolin 7-*O*-glucoside ([M-H]^-^447.09328) and naringenin isomers ([M-H]^-^ 433.11402) were the unique identified compounds in the flavone and flavanones sub-classes, respectively. Within the flavonol sub-class, quercetin, isorhamnetin, and kaempferol glycosides, were identified with diagnostic ions at 301, 315 and 285, respectively. Moving to positive ion mode detection, the largest sub-class comprised anthocyanins and anthocyanidins, with cyanidin, peonidin, pelargonidin, delphinidin and their glycosides as key markers (Martini et al., 2017).

**Figure 4:**
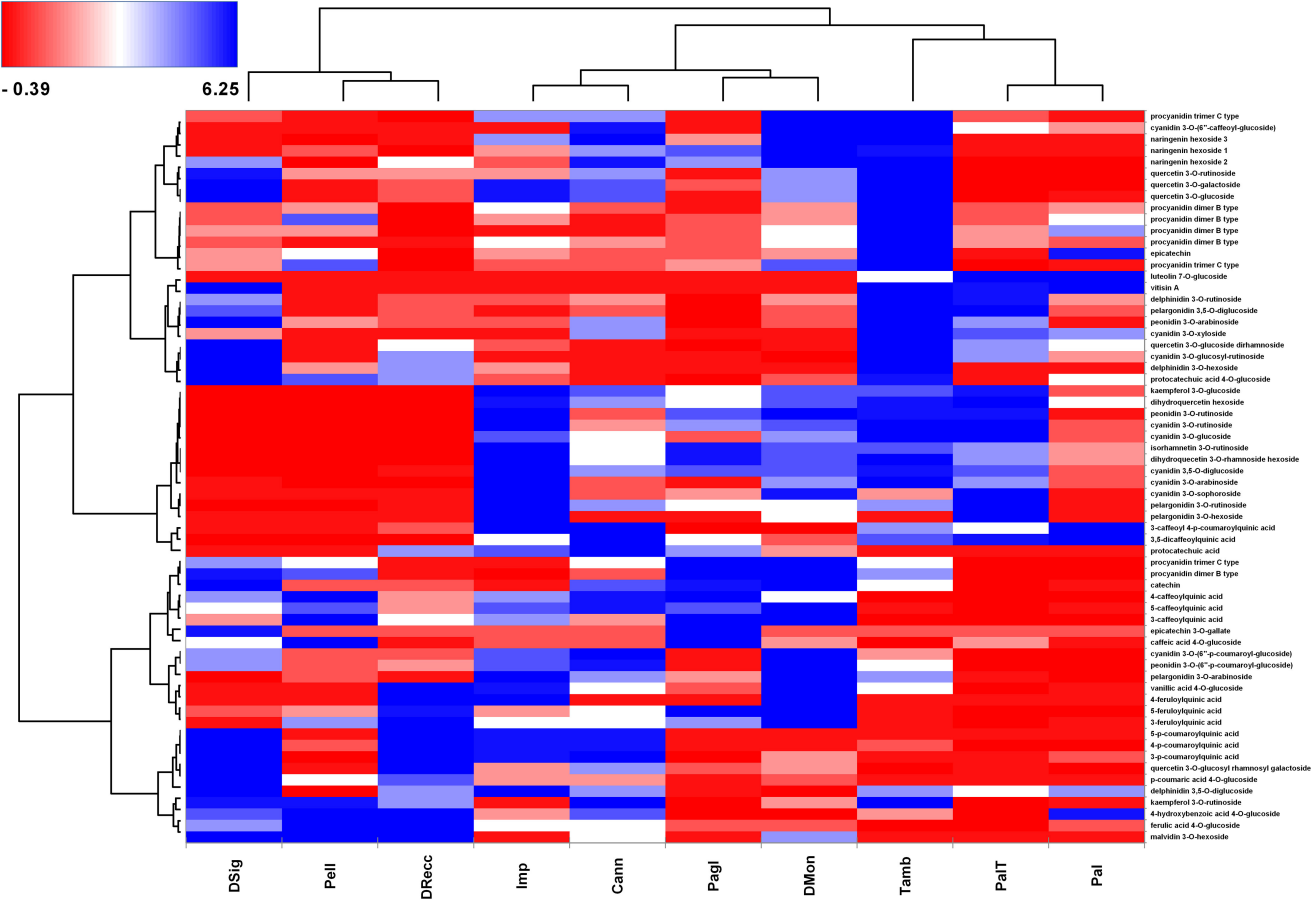
Heat-map reporting the variable abundance of polyphenols in sweet cherry cultivars. Each column corresponds to a cultivar, whereas each row represents a phytochemical upon manual curation of tandem MS spectra. Heat-map encompassed centered and reduced intensities (area counts) moving from red to blue, through white, including Euclidean distance function and Ward linkage method. The dendrograms from the hierarchical cluster analysis of the columns and the rows illustrate the similarity of the cultivars and the distribution of secondary metabolites, respectively.

**Figure 4** outlines the molecular (y axis) and cultivar (x axis) grouping based on hierarchical clustering with the Ward linkage method. The color scale ranges from red to blue through white, with centered and scaled values of polyphenol area counts in cherry samples. In line with the proteomic analysis, we observed three distinct groups. In the left-down corner, the accumulation of malvidin 3-*O*-hexoside, ferulic acid 4-*O*-glucoside, 4-hydroxybenzoic acid 4-*O*-glucoside and kaempferol 3-O-rutinosided represented the typical molecular signature of DSig, Pell and DRecc cultivars (group 3). Conversely, the concentration of these compounds was lower in the other seven cultivars. Molecular grouping based on caffeoylquinic acid and feruloylquinic acid derivatives characterized Imp, Cann, Pagl, and DMon accessions (group 1). The top-right corner included compounds part of procyanidin dimer B type and trimer C type, naringenin and cyanidin derivatives, which clustered Tamb, PalT, and Pal ecotypes (group 2).

**Figure 5:**
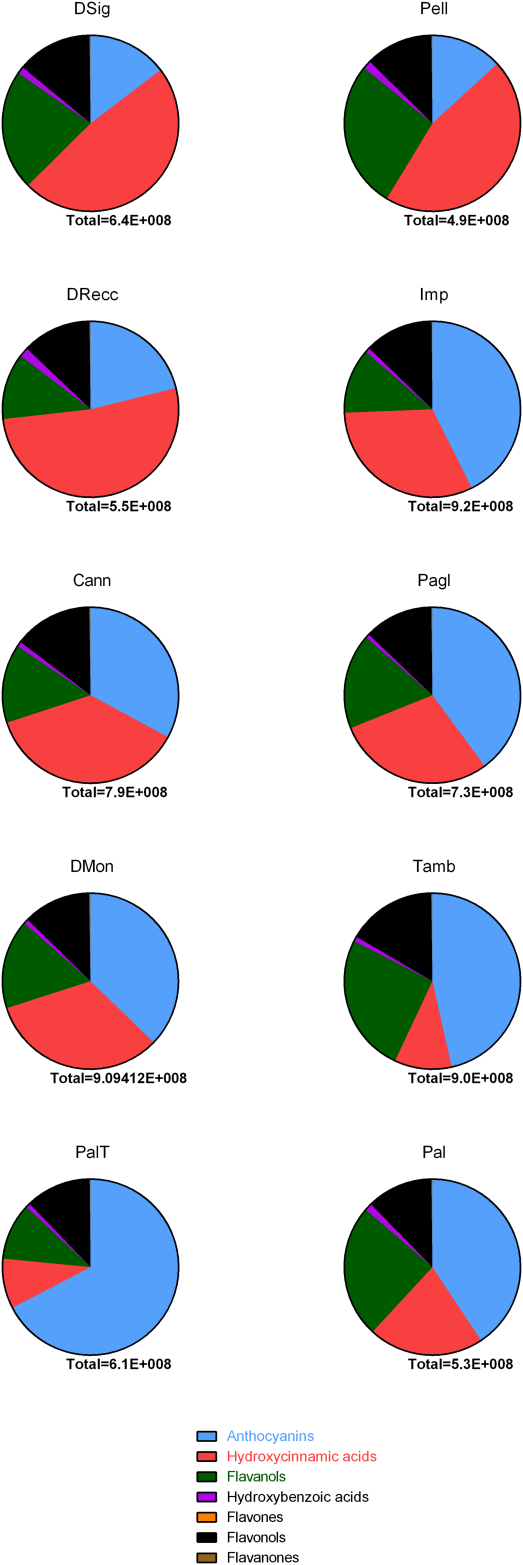
Pie-chart summarizing the distribution of polyphenol classes in sweet cherry accessions according to publicly available databases. Each compounds identified was included in the following families using as a reference area counts of full MS signals in FTMS mode: anthocyanins, hydroxycinnamic acids, flavanols, hydroxybenzoic acids, flavones and flavanones in line with phenol-explorer database.

The heatmap served as the basis for a more accurate cultivar classification based on polyphenol subclasses, as depicted in pie-charts in **Figure 5**. The area counts of each polyphenol contributed to the distribution in percentage of different polyphenol subclasses, providing information on the spatial distribution and area-coverage according to the heatmap in **Figure 4**. Across all cultivars, the average concentration of hydroxybenzoic acids, flavanones, flavonols and flavones remained similar. However, significant changes in specific secondary metabolites were observed for anthocyanins, hydroxycinnamic acids and flavanols. The first cluster (DSig, Pell and DRecc – group 3) was characterized by a neat prevalence of hydroxycinnamic acids (47.9%, 45.5% and 52.1%, respectively), while anthocyanins did not exceed 21.1% (DRecc). Additionally, two out of three cultivars (Pell and DSig) in this cluster exhibited a high percentage of flavanols (27.2% and 22.1%, respectively). The second cluster, including Imp, Cann, Pagl and DMon accessions (group 1), envisaged increasing concentrations of anthocyanins that anyway remained lower than those of the third cluster. Specifically, the percentage of anthocyanins was 42.6%, 32.9%, 39.9% and 37.3% in Imp, Cann, Pagl and DMon cultivars, respectively. The third cluster, encompassing Tamb, PalT and Pal accessions (group 2), exhibited the highest percentage of anthocyanins; this finding was associated with the lowest percentage of hydroxycinnamic acids.

### 3.5 Anthocyanin biosynthesis enzymes reveal cultivar-specific signatures in sweet cherry

To verify the linear relationship between the 63 DRPs and the 64 metabolites analyzed in the study, a Pearson correlation analysis (p<0.01) was carried out (**Supplementary Figure S5**). A marked positive correlation was observed between nine metabolites (cyanidin 3-*O*-rutinoside, cyanidin 3-*O-*sophoroside, cyanidin 3,5-*O*-diglucoside, pelargonidin 3-*O*-rutinoside, peonidin 3-*O*-rutinoside, dihydroquecetin 3-*O*-rhamnoside hexoside, dihydroquercetin hexoside, isorhamnetin 3-*O*-rutinoside, kaempferol 3-*O*-glucoside), primarily anthocyanidins and flavonols, and up to ten enzymes associated with flavonoid metabolism (DFR, UFGT, C4H1, CHS1, F3H1, ANS, CHI, CHI, CYP75B1, UGT1). This outcome validates the results of the hierarchical clustering analysis of 63 DRPs (**Figure 3**), which highlighted the enzymes involved in the flavonoid biosynthetic process as the proteins with the highest variability among the ten cultivars holding the greatest discriminative power.

**Figure 6:**
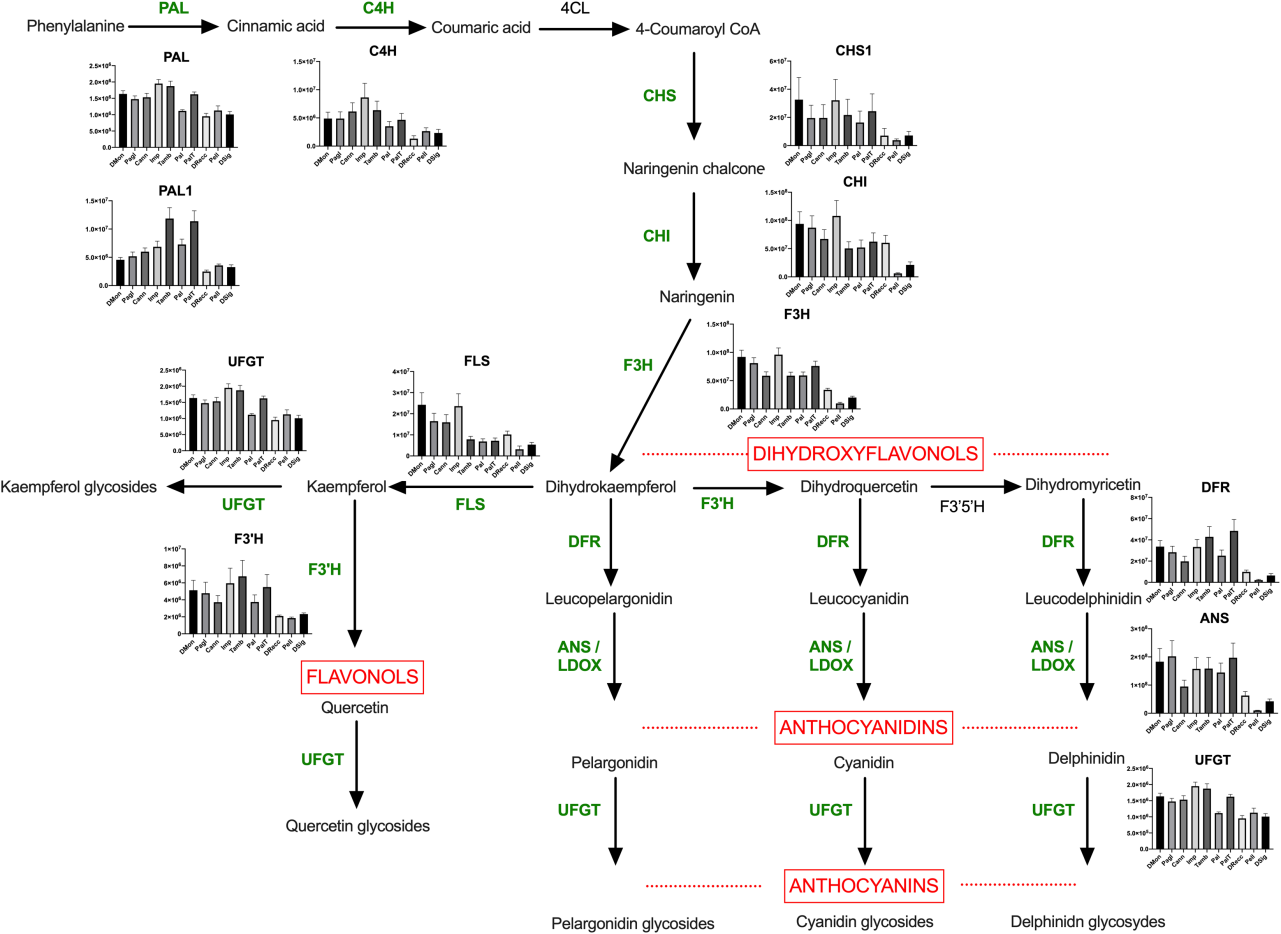
Anthocyanin pathway in sweet cherry. The enzymes identified and quantified in this study have been reported in green. Abbreviations are as follows: PAL, phenylalanine ammonialyase; PAL1, phenylalanine ammonialyase; C4H, cinnamate 4-hydroxylase; 4CL, 4-coumarate-CoA ligase; CHS, chalcone synthase; CHI, chalcone isomerase; F3’H, flavonoid 3’-hydroxylase; F3’5’H, flavonoid 3’,5’-hydroxylase; F3H, flavonoid 3-hydroxylase; DFR, dihydroflavonol 4-reductase; FLS, flavonol synthase; ANS, anthocyanidin synthase; UFGT, UDPglucose flavonoid 3-O-glucosyl transferase. Differences among cultivars for each enzyme were analyzed by multiple comparisons through one-way ANOVA (α = 0.05) and the results have been reported in **Supplementary Table S6**.

To visually represent the abundance of the proteins involved in the flavonoid biosynthesis in all ecotypes, we mapped them within the biosynthetic pathway of anthocyanins (**Figure 6**). All the enzymes displayed lower abundance values in DRecc, Pell, and DSig cultivars (group 3), compared to DMon, Pagl, Cann, Imp (group 1) and Tamb, Pal, and PalT accessions (group 2). Multiple comparisons through one-way ANOVA with Tukey HSD test analyzed the differences in the enzyme abundances among the accessions, using a significance level α of 0.05. Detailed results are provided in **Supplementary Table S6**. Additionally, Pearson correlation analysis was performed to investigate the relationship between the relative content of the enzymes of the biosynthetic pathway of flavonoids and the quantitative levels of anthocyanins, as estimated from area counts of tandem mass spectrometry experiments (**Supplementary Figure S6**). This analysis corroborated the distribution of the sweet cherry cultivars into the three groups already defined according to both proteomic and metabolomic data; the unique exception was the ecotype Pal, which exhibited abundance values lower than that of the other cultivars in the same group (Tamb, and PalT). Furthermore, a strong correlation with the content of anthocyanins was observed for two enzymes, namely dihydroflavonol 4-reductase (DFR) and flavonoid 3’-hydroxylase (F3’H) (r>0,8), while the correlation for the enzymes flavonol synthase (FLS) and chalcone isomerase (CHI) was not statically significant.

## 4 Discussion

*P. avium* genetic diversity has been studied using rapid techniques for DNA fingerprinting that assessed proper parameters for differentiation of European and American ecotypes, assigning ancestral plant populations and identifying germplasm redundancies (Barreneche et al., 2021; Muccillo et al., 2019). For example, using SSR markers, Muccillo and coworkers have genotyped sweet cherry cultivars from the Campania region, revealing a distinct genetic constitution for regional cultivars compared to commercial ones (Muccillo et al., 2019). In parallel, phenotypic characterization of Italian and Campania sweet cherry accessions defined differences in fruit chemical composition, nutritional value, and antioxidant properties (Berni et al., 2021; Clodoveo et al., 2023; Martini et al., 2019; Pacifico et al., 2014), suggesting that their phenolic content is primarily determined by the genetic background of plants.

Recent years have witnessed the application of proteomics in elucidating different aspects of sweet cherry physiology, including fruit development, ripening, and abscission (Michailidis et al., 2020b; Prinsi et al., 2016; Qiu et al., 2020). Notably, the proteogenomic spatial description has emerged as an important system biology approach leading to the development of a comprehensive sweet cherry atlas through the analysis of 15 tissues (Xanthopoulou et al., 2022). However, only a few efforts have been devoted so far to the characterization of the phenotypic diversity of cherry varieties through a proteomic approach (Berni et al., 2021). In the present study, we have used an integrated proteo-metabolomic approach to characterize sweet cherry cultivars from the Campania region, aiming at comprehensive molecular phenotyping of the corresponding fruits. Exploiting a gel-free TMT-based procedure for the first time in this context, we have identified a total of 3786 proteins in sweet cherry accessions, among which 288 DRPs in at least one pairwise comparison. This approach demonstrated enhanced analytical sensitivity compared to the gel-based methodology previously used by Berni and coworkers for the characterization of various plant ecotypes from Tuscany (Berni et al., 2021). Further analysis utilizing KO assignment and KEGG Pathway reconstruction functions allowed the identification of 63 DRPs involved in the biosynthesis of secondary metabolites. Their relative abundance proved instrumental in differentiating sweet cherry cultivars. Concurrently, the study of phytochemicals part of polyphenol families pinpointed anthocyanins as a key class for phenotypic differentiation of sweet cherry ecotypes. Cultivar grouping based on metabolite abundance yielded similar results to those referred to the relative abundance of DRPs, effectively dividing the investigated sweet cherry accessions into three parallel groups. In this respect, concomitant identification and quantification of polyphenols as well as of enzymes involved in their biosynthesis provided important information on the dynamic processes regulated by the genetic background of sweet cherries, in agreement with other studies on other plant cultivars (Mozetič et al., 2004).

Extensive research has been conducted in sweet cherry on genes implicated in fruit coloring development to investigate the corresponding expression during fruit ripening (Chen et al., 2022; Mozetič et al., 2004). A transcriptomic study comparing dark red and yellow sweet cherry cultivars provided valuable insights into the contribution of the anthocyanin biosynthetic pathway in fruit coloration, defining the differential expression of the genes PAL, 4CL, CHS, CHI, F3H, F3’H, DFR, ANS, and UFGT in various ecotypes (Wei et al., 2015). In this study, the group including DRecc, Pell, and DSig cultivars exhibited notably low levels of total anthocyanins and demonstrated diminished abundance of most of the enzymes involved in flavonoid metabolism, crucial as anthocyanin precursors. These accessions were also characterized by lighter peel and pulp colors as evidenced by the highest values of color indexes L*, a*, and b*. These phenotypic results demonstrated the strong correlation observed in this case between color indexes and the abundance of total anthocyanins and enzymes involved in flavonoid metabolism, confirming the above-mentioned transcriptomic outcomes. However, the same correlation with color indexes was not observed for the remaining seven cultivars. Specifically, the ecotype Imp, despite displaying intermediate color indexes, ranked third in terms of total anthocyanins and exhibited among the highest abundance of enzymes associated with flavonoid metabolism. Regarding the group comprising Tamb, Pal, and PalT cultivars, high levels of anthocyanins were detected in Tamb and PalT, while Pal exhibited lower anthocyanin levels. This pattern closely mirrored that of the enzyme UFGT involved in the anthocyanidin glycosylation, along with the trend observed for several other precursor enzymes of flavonoid metabolism, such as PAL, C4H, CHS1, F3’H, DFR. The observations reported above suggested that other compounds in addition to anthocyanins should probably contribute to the coloration of the dark sweet cherries here investigated. Recently, tannic, caffeic, 4-hydroxybenzoic, gallic, and malic acids and other polymer forms have been demonstrated highly affecting the anthocyanin-based pigmentation of sour cherry (*P. cerasus* L.) (Molaeafard et al., 2021). Accordingly, different levels of these organic acids in DMon, Pagl, Cann, Imp, Tamb, Pal and PalT cultivars may hypothetically influence the color of these cherries, justifying the corresponding absence of correlation between color indexes and the abundance of anthocyanins and enzymes involved flavonoid biosynthesis.

Altogether, the color and proteo-metabolic differences here described for the above-reported Campania cultivars are not surprising, since they find a parallel in results from previous investigations on other Italian, European and Chinese red and yellow sweet cherry ecotypes (Berni et al., 2021; Guo et al., 2018; Wei et al., 2015; Zhai et al., 2022), which reported a high variability in anthocyanin-related metabolites and enzymes in the investigated accessions. In these cases, multi-omic technologies associated the observed phenotypic differences with cultivar-specific expression of genes encoding for enzymes and transcription factors involved in anthocyanin biosynthesis as well as in abscisic acid and gibberellic acid signaling pathways, suggesting that light-dependent molecular mechanisms might be related to color and polyphenol content ecotype variances. These findings found a parallel with high-resolution genome-wide association studies on large European sweet cherry collections that identified a high frequency of deletions or SNPs associated with fruit color in chromosome 3 (Holušová et al., 2023; Calle et al., 2021). The affected chromosome portion was demonstrated to contain five examples of MYB proteins that were already described as major modifiers of anthocyanin biosynthesis (Jin et al., 2016; Holušová et al., 2023). By exploiting advanced analytical techniques and statistical analyses, the present proteo-metabolomic study thus discerned subtle differences between Campania cultivars, contributing to their molecular phenotyping. Our findings underscored the complex interplay between genetic regulation, enzyme activity, metabolite representation and fruit coloration in sweet cherry ecotypes. To further validate and expand these results, future studies integrating functional proteomics with genomics and transcriptomics would allow for a more comprehensive understanding of the specific roles exerted by the identified proteins and the genetic regulation underlying the observed metabolic variations among fruit ecotypes. This integrative, multi-omics strategy could provide valuable insights into the molecular mechanisms driving ecotype-specific quality traits contributing to the identification of key biomarkers to facilitate cultivar selection and targeted breeding strategies aimed at improving fruit color, flavor, and nutritional value of sweet cherry.

By elucidating the protein profiles of these accessions, we also provided deeper insights into biochemical composition of sweet cherry accessions in terms of allergenic proteins, describing their variability. The TMT-based proteomic approach entailed the identification of several protein sequences corresponding to major allergens in sweet cherry fruits, providing information on the contribution of different isoforms to the total allergenic potential of each ecotype. The ability to accurately characterize and differentiate cultivars at this molecular level may be potentially used in the next future to develop dedicated interventions aimed at selecting specific accessions to meet consumer demand for low allergenic fruits or related food products. The untargeted approach adopted in the present study for proteomic and metabolomic experiments was intentional as it enabled an early-stage comprehensive comparison of the investigated sweet cherry ecotypes, offering identification and relative quantification of thousands of molecules, and fostering biomarker discovery and data-driven hypothesis generation. Similarly to other untargeted studies, this investigation should hypothetically suffer from the lack of absolute molecular quantification, which enhances measurement precision and avoids the possible occurrence of analytical inconsistencies. These limits are generally overcome by targeted experiments, which are preferred for research validation and implementation, and concentrate on a known set of proteins and metabolites precisely quantifying them. In the next future, targeted proteomic and metabolomic experiments could tackle the composition and distribution of selected metabolite and protein markers within cherry peel and skin, potentially tailoring the biodiversity of selected cultivars to improve the accumulation of taste- or health-effective compounds, and the study of post-harvest effects on fruit organoleptic and quality attributes (Chockchaisawasdee et al., 2016).

## 5 Conclusion

Our integrated proteo-metabolomic approach provided comprehensive insights into the molecular characteristics of sweet cherry cultivars from the Campania region elucidating coherent phenotypic relationships and associations between them. Statistical analyses played a crucial role in our study, enabling the detection and interpretation of even minimal differences between ecotypes. By using rigorous mass spectrometry techniques combined with chemometrics, multivariate data analysis and statistical methods, we could effectively highlight the molecular peculiarities of each cultivar, contributing to a comprehensive understanding of sweet cherry biodiversity, thus elucidating their unique traits and potential applications. Our findings highlight the biochemical diversity among sweet cherry cultivars, underscoring the importance of molecular characterization in fruit breeding programs and horticultural practices. The ability to accurately characterize and differentiate fruit cultivars at the molecular level, through integrated proteins and metabolites analyses, will facilitate dedicated targeted breeding, quality control, and cultivar selection efforts to meet consumer preferences and market demands.

## Data Availability

The datasets presented in this study can be found in online repositories. The names of the repository/repositories and accession number(s) can be found in the article/**Supplementary Material**.
