# Supplementary figures and images for: Proteo-metabolomic analysis of fruits reveals molecular insights into variations among Italian Sweet Cherry (Prunus avium L.) accessions
Source: Front Plant Sci. 2025 Jun 3;16:1591996. doi: 10.3389/fpls.2025.1591996 (PMC12170513; doi:10.3389/fpls.2025.1591996)

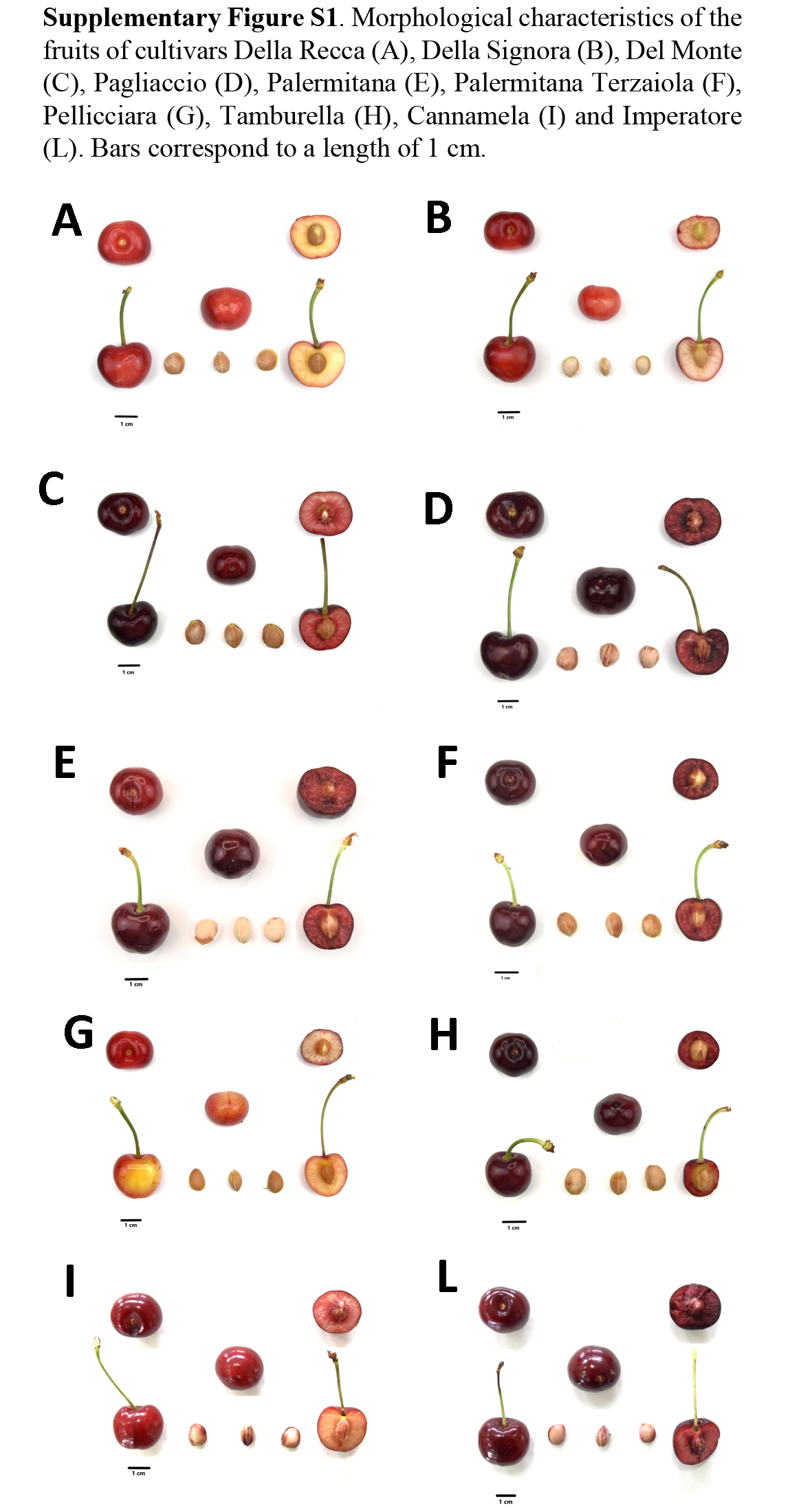

Supplement: Supplementary file 1 [file Image1.tif]

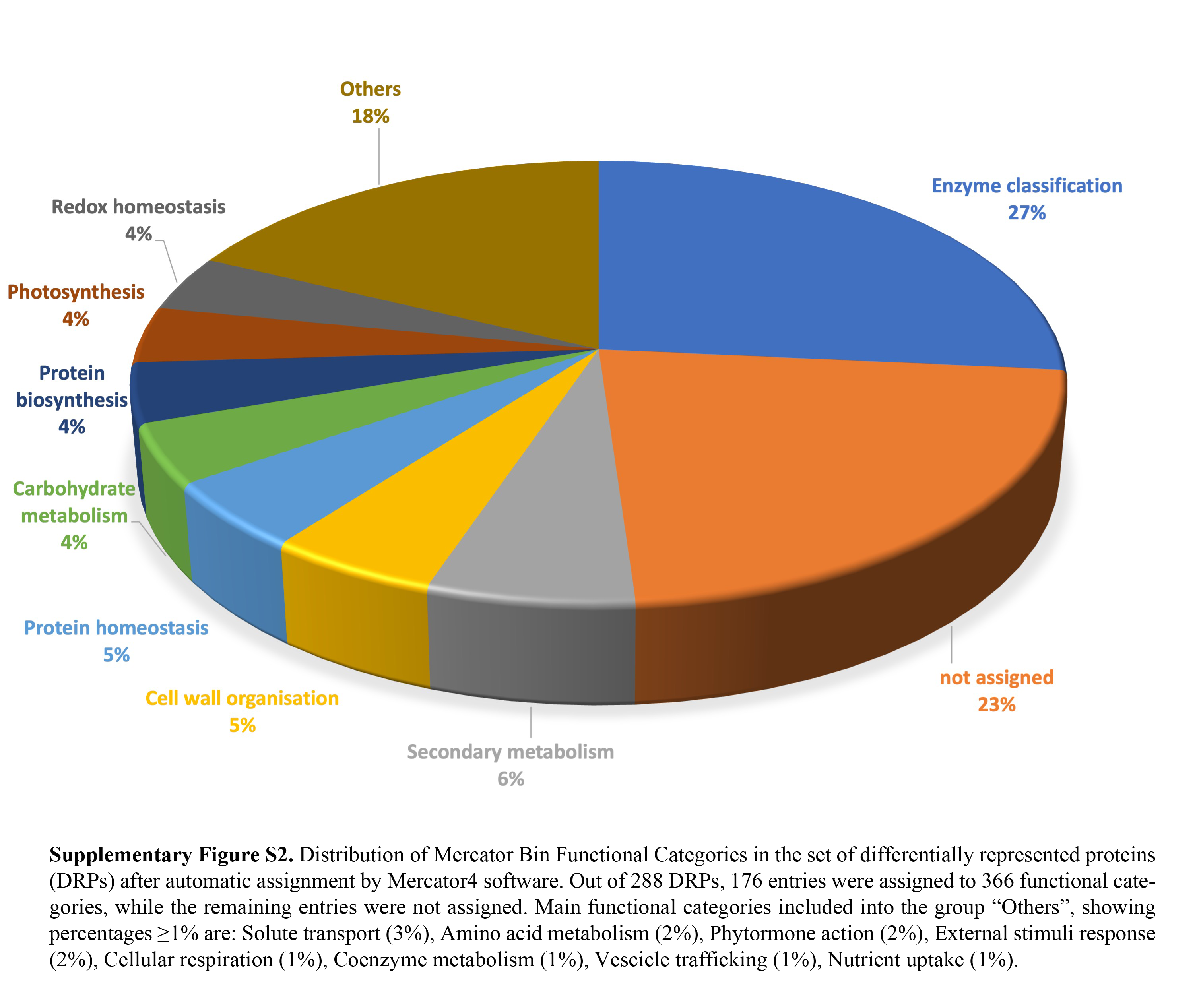

Supplement: Supplementary file 2 [file Image2.tif]

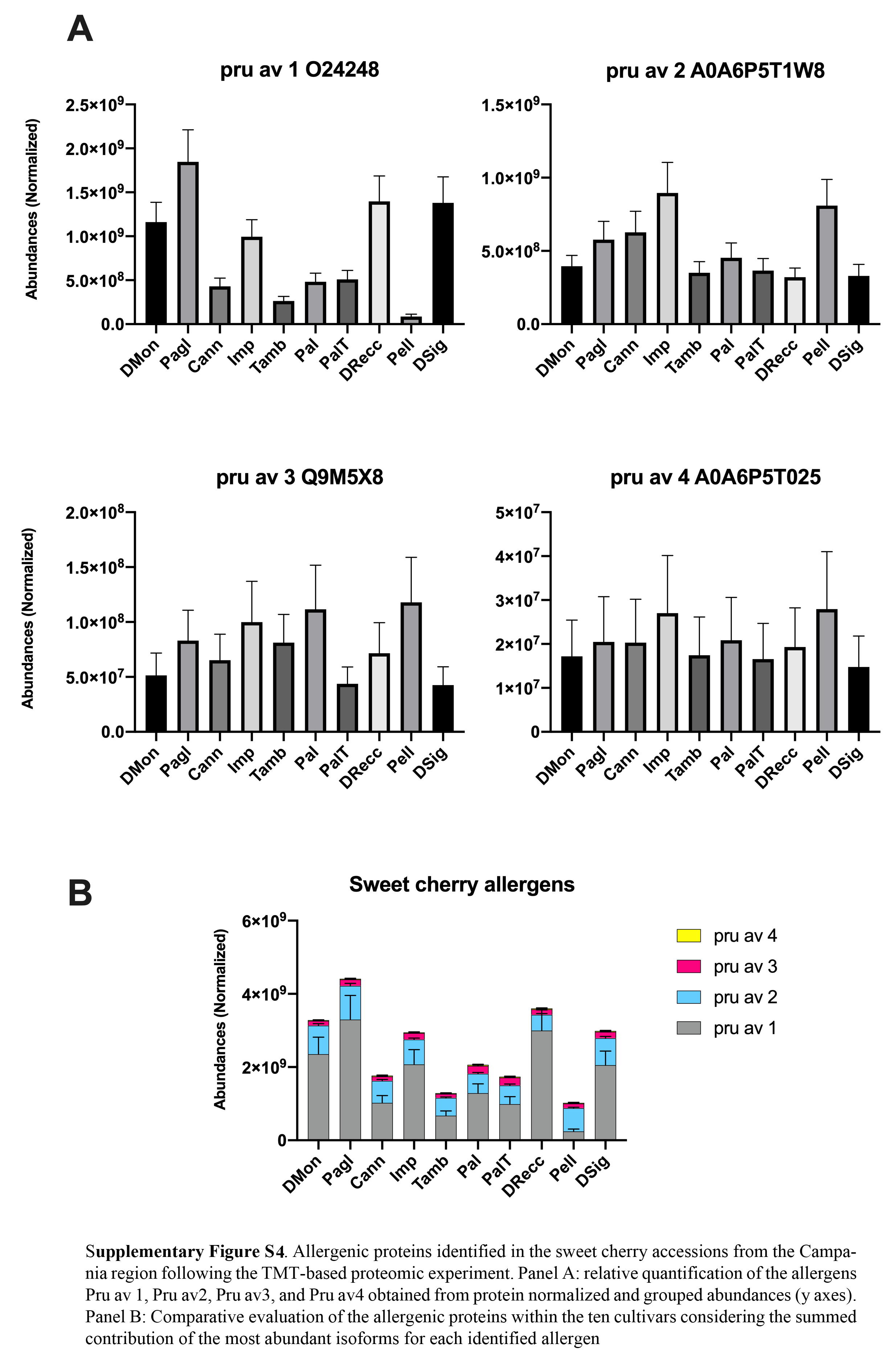

Supplement: Supplementary file 4 [file Image4.tif]

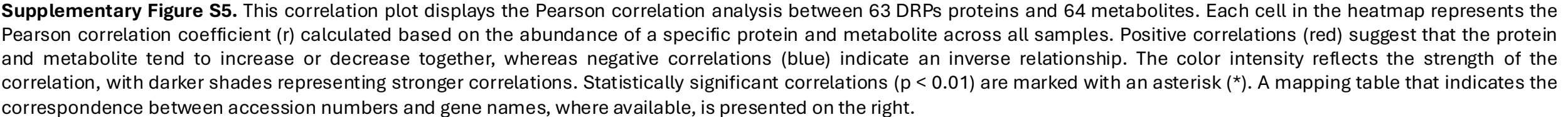

Supplement: Supplementary file 5 [file Image5.pdf]
