# Supplementary material for: Proteo-metabolomic analysis of fruits reveals molecular insights into variations among Italian Sweet Cherry (Prunus avium L.) accessions
Source: Front Plant Sci. 2025 Jun 3;16:1591996. doi: 10.3389/fpls.2025.1591996 (PMC12170513; doi:10.3389/fpls.2025.1591996)
Supplement: Supplementary file 3 [file Image3.pdf]

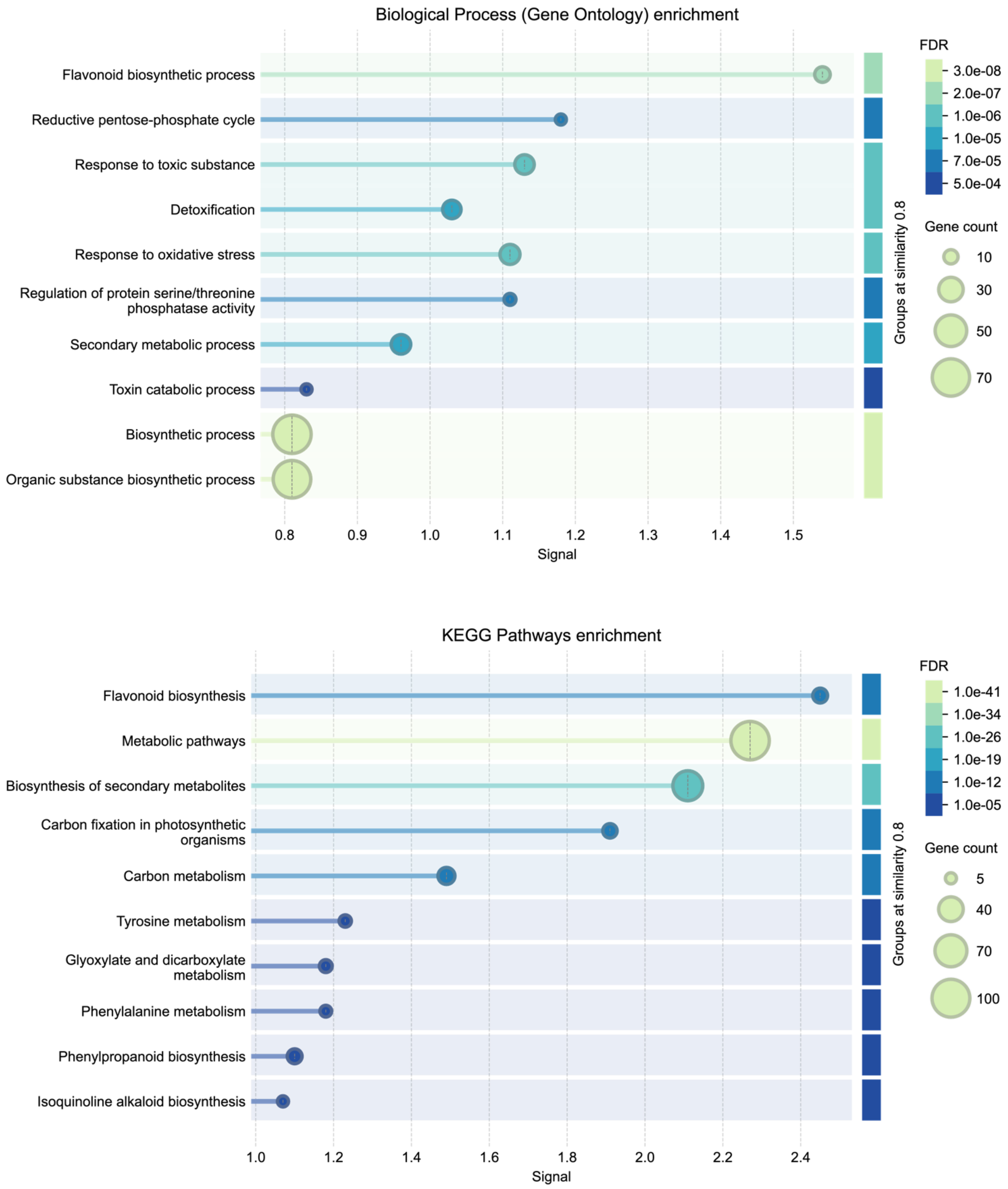

Supplementary Figure S3. Overview of the functional enrichment analysis for the differentially represented proteins (DRPs). The DRPs were selected if they significantly changed in at least one by one comparison between cultivars (p-value ≤ 0.05 with log2FC ≥ 1.0 or log2FC ≤ -1.0). Enrichment of the GO -biological process terms (A) and KEGG pathway terms (B). The analysis was obtained through STRING (https://string-db.org); the statistical significance of enrichment was assessed using the built-in false discovery rate (FDR) correction for multiple testing (Benjamini-Hochberg procedure) and were reported ordering for the "signal" parameter, which is defined as a weighted harmonic mean between the observed/expected ratio and -log(FDR).
