# Supplementary material for: Proteo-metabolomic analysis of fruits reveals molecular insights into variations among Italian Sweet Cherry (Prunus avium L.) accessions
Source: Front Plant Sci. 2025 Jun 3;16:1591996. doi: 10.3389/fpls.2025.1591996 (PMC12170513; doi:10.3389/fpls.2025.1591996)
Supplement: Supplementary file 6 [file Image6.pdf]

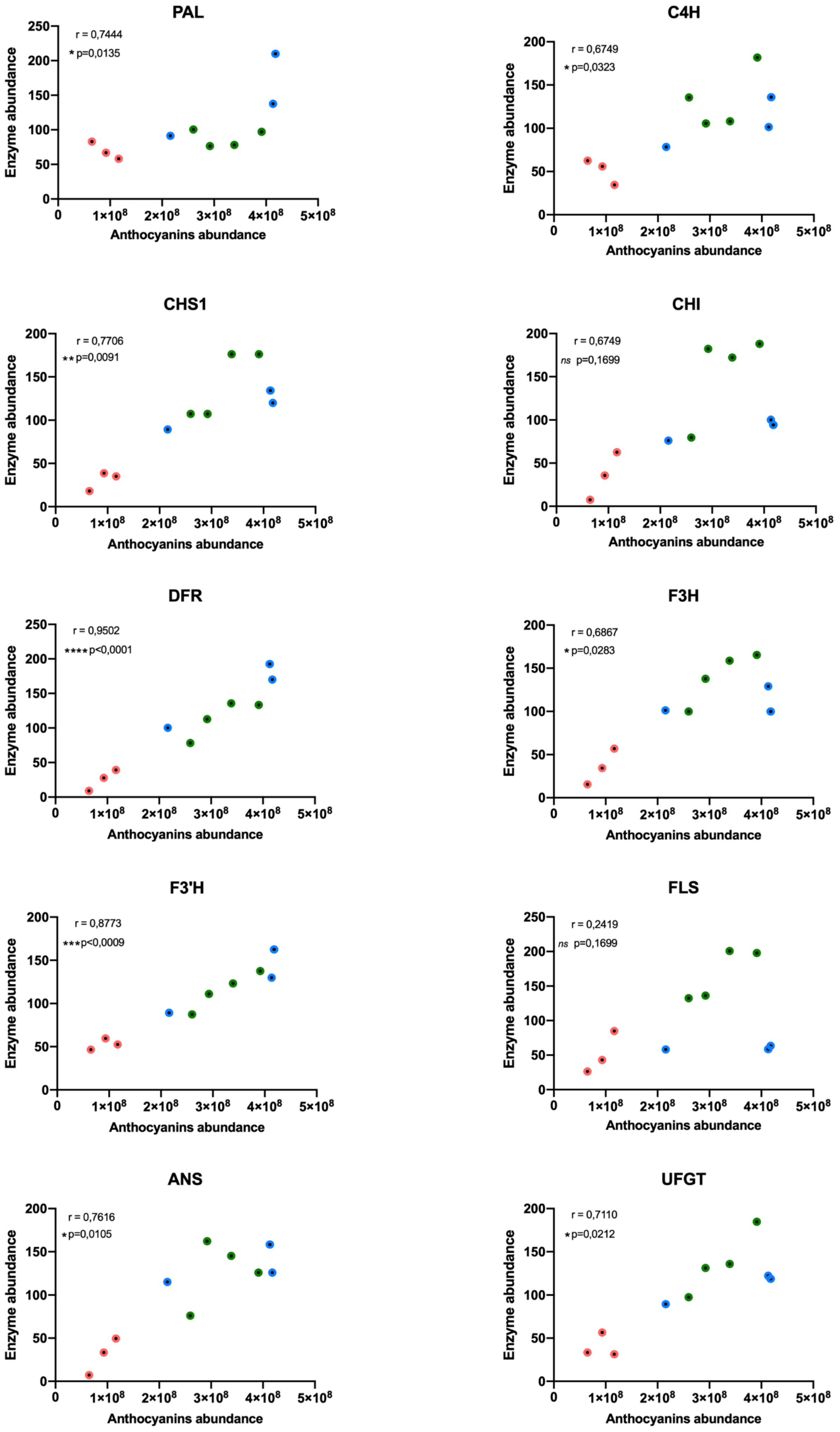

Supplementary Figure S6. Pearson correlation analysis of the abundance of the enzymes involved in flavonoid metabolism measured in this study vs. total anthocyanins content determined from LC-MS/MS area counts of all identified anthocyanins. Abbreviations are as follows: PAL, phenylalanine ammonia lyase; C4H, cinnamate 4-hydroxylase; CHS1, chalcone synthase 1; CHI, chalcone isomerase; DFR, dihydrofavonol 4-reductase; F3H, favonoid 3-hydroxylase; F3'H, favonoid 3'- hydroxylase; FLS, favonol synthase; ANS, anthocyanidin synthase; UFGT, UDPglucose flavonoid 3-O-glucosyl transferase. Red spots: cultivars Della Recca (DRecc), Pellicciara (Pell), and Della Signora (DSig). Green spots: cultivars Del Monte (DMon), Pagliaccio (Pagl), Cannamela (Cann), and Imperatore (Imp). Blue spots: cultivars Tamburella (Tamb), Palermitana (Pal), and Palermitana Terzaiola (PalT). Pearson's correlation coefficient (r) and p-value (p) have been reported in the graphs.
