# Supplementary material for: Proteo-metabolomic analysis of fruits reveals molecular insights into variations among Italian Sweet Cherry (Prunus avium L.) accessions
Source: Front Plant Sci. 2025 Jun 3;16:1591996. doi: 10.3389/fpls.2025.1591996 (PMC12170513; doi:10.3389/fpls.2025.1591996)
Supplement: Supplementary file 7 [file Table5.docx]

**Supplementary Table S5. Polyphenols identified in positive [M]^+^ and negative mode [M-H]^-^ in sweet cherry fruits.** Polyphenols were included in different sub-classes according to Phenol-explorer database. Experimental mass values were obtained with an accuracy of ± 5 ppm, with respect to theoretical counterparts. Compounds identification was achieved in data-dependent scanning mode with specific relative intensities.

|  | **Compound** | **Theoretical mass [M-H]^-^** | **Theoretical mass [M]^+^** | **Accuracy (ppm)** | **MS/MS main ion fragments (m/z)** |
| --- | --- | --- | --- | --- | --- |
| **Hydroxycinnamic acids** | |  |  |  |  |
|  | 3-caffeoylquinic acid | 353.08781 |  | -0.57 | 191, 179, 135 |
|  | 5-caffeoylquinic acid | 353.08781 |  | -0.63 | 191, 179, 135 |
|  | 4-caffeoylquinic acid | 353.08781 |  | -0.77 | 191, 179, 135 |
|  | 3-feruloylquinic acid | 367.10346 |  | 0.35 | 193, 134 |
|  | 5-feruloylquinic acid | 367.10346 |  | 0.67 | 193, 134 |
|  | 4-feruloylquinic acid | 367.10346 |  | 1.51 | 193, 134 |
|  | 3-p-coumaroylquinic acid | 337.09289 |  | 0.69 | 163, 119, 191 |
|  | 4-p-coumaroylquinic acid | 337.09289 |  | 0.73 | 163, 119, 191 |
|  | 5-p-coumaroylquinic acid | 337.09289 |  | 0.68 | 163, 119, 191 |
|  | 3,5-dicaffeoylquinic acid | 515.11950 |  | 1.35 | 353, 191 |
|  | p-coumaric acid 4-O-glucoside | 325.09289 |  | 3.15 | 163, 187 |
|  | caffeic acid 4-O-glucoside | 341.08780 |  | -2.29 | 179, 135 |
|  | ferulic acid 4-O-glucoside | 355.10346 |  | 1.81 | 193, 217, 175 |
|  | 3-caffeoyl 4-p-coumaroylquinic acid | 499.12458 |  | 1.21 | 337, 353, 173 |
| **Hydroxybenzoic acids** | |  |  |  |  |
|  | 4-hydroxybenzoic acid 4-O-glucoside | 299.07724 |  | 1.50 | 137 |
|  | vanillic acid 4-O-glucoside | 329.08781 |  | -1.37 | 167,152 |
|  | protocatechuic acid | 153.01933 |  | 2.74 | 109 |
|  | protocatechuic acid 4-O-glucoside | 315.07216 |  | 1.27 | 153, 109 |
| **Flavanols** | |  |  |  |  |
|  | procyanidin dimer B type | 577.13515 |  | 1.01 | 425, 407, 289 |
|  | procyanidin dimer B type | 577.13515 |  | 1.05 | 425, 407, 289 |
|  | procyanidin dimer B type | 577.13515 |  | 1.21 | 425, 407, 289 |
|  | procyanidin dimer B type | 577.13515 |  | 1.10 | 425, 407, 289 |
|  | procyanidin dimer B type | 577.13515 |  | 1.00 | 425, 407, 289 |
|  | procyanidin trimer C type | 865.19854 |  | 1.35 | 695, 577, 739, 713 |
|  | procyanidin trimer C type | 865.19854 |  | 1.30 | 695, 577, 739, 713 |
|  | procyanidin trimer C type | 865.19854 |  | 1.21 | 695, 577, 739, 713 |
|  | catechin | 289.07176 |  | 2.01 | 245, 205, 179 |
|  | epicatechin | 289.07176 |  | 0.95 | 245, 205, 179 |
|  | epicatechin 3-O-gallate | 441.08272 |  | 0.85 | 330, 397, 161 |
| **Flavones** | |  |  |  |  |
|  | luteolin 7-O-glucoside | 447.09328 |  | 0.93 | 285 |
| **Flavonols** | |  |  |  |  |
|  | quercetin 3-O-glucosyl rhamnosyl galactoside | 771.19893 |  | 2.21 | 609, 463 |
|  | quercetin 3-O-glucoside dirhamnoside | 755.20402 |  | 3.17 | 593,447 |
|  | quercetin 3-O-rutinoside | 609.14611 |  | 2.31 | 301, 271 |
|  | quercetin 3-O-glucoside | 463.08820 |  | 2.57 | 301 |
|  | quercetin 3-O-galactoside | 463.08820 |  | 1.29 | 301 |
|  | dihydroquercetin hexoside | 465.10385 |  | -1.20 | 285, 241, 339 |
|  | dihydroquecetin 3-O-rhamnoside hexoside | 611.16176 |  | -0.91 | 285, 485, 475, 501, 241, 303 |
|  | kaempferol 3-O-rutinoside | 593.15119 |  | 1.35 | 285, 561, 253 |
|  | kaempferol 3-O-glucoside | 447.09328 |  | 1.27 | 285, 327, 255 |
|  | isorhamnetin 3-O-rutinoside | 623.16176 |  | 1.21 | 315, 300 |
| **Flavanones** | |  |  |  |  |
|  | naringenin hexoside 1 | 433.11402 |  | 0.95 | 271, 151, 313 |
|  | naringenin hexoside 2 | 433.11402 |  | 0.65 | 271, 151, 313 |
|  | naringenin hexoside 3 | 433.11402 |  | 0.47 | 271, 151, 313 |
| **Anthocyanins** | |  |  |  |  |
|  | cyanidin 3-O-(6''-caffeoyl-glucoside) |  | 611.13953 | 1.21 | 449, 287 |
|  | peonidin 3-O-(6''-p-coumaroyl-glucoside) |  | 609.16027 | 1.19 | 301 |
|  | cyanidin 3-O-glucoside |  | 449.10784 | -075 | 287 |
|  | peonidin 3-O-arabinoside |  | 433.11292 | -1.91 | 301 |
|  | cyanidin 3-O-(6''-p-coumaroyl-glucoside) |  | 595.14461 | 0.85 | 433, 287 |
|  | cyanidin 3-O-rutinoside |  | 595.16574 | 0.93 | 449, 287 |
|  | vitisin A [Malvidin 3-O-glucoside pyruvic acid] |  | 561.12388 | 2.10 | 399 |
|  | malvidin 3-O-hexoside |  | 493.13405 | 1.51 | 331 |
|  | pelargonidin 3-O-hexoside |  | 433.11292 | 2.63 | 271 |
|  | cyanidin 3-O-xyloside |  | 419.09727 | 1.98 | 287 |
|  | pelargonidin 3-O-rutinoside |  | 579.17083 | 1.96 | 271 |
|  | peonidin 3-O-rutinoside |  | 609.18140 | 0.95 | 463,301 |
|  | cyanidin 3-O-arabinoside |  | 419.09727 | -0.85 | 287 |
|  | pelargonidin 3-O-arabinoside |  | 403.10236 | 0.57 | 271 |
|  | cyanidin 3-O-glucosyl-rutinoside |  | 757.21857 | 1.53 | 433, 287 |
|  | delphinidin 3,5-O-diglucoside |  | 627.15558 | 2.10 | 465, 303 |
|  | cyanidin 3,5-O-diglucoside |  | 611.16066 | 1.23 | 449, 287 |
|  | cyanidin 3-O-sophoroside |  | 611.16066 | 0.99 | 287 |
|  | pelargonidin 3,5-O-diglucoside |  | 595.16575 | 0.97 | 433, 271 |
|  | delphinidin 3-O-hexoside |  | 465.10275 | -1.3 | 303 |
|  | delphinidin 3-O-rutinoside |  | 611.16066 | 0.67 | 303, 465 |
